# ERK1 and ERK2 present functional redundancy in tetrapods despite higher evolution rate of ERK1

**DOI:** 10.1186/s12862-015-0450-x

**Published:** 2015-09-03

**Authors:** Roser Buscà, Richard Christen, Matthew Lovern, Alexander M. Clifford, Jia-Xing Yue, Greg G. Goss, Jacques Pouysségur, Philippe Lenormand

**Affiliations:** Institute for Research on Cancer and Aging of Nice (IRCAN), University of Nice-Sophia Antipolis, CNRS UMR7284, INSERM, Centre A. Lacassagne, Nice, 06189 France; CNRS UMR 7138, Systématique-Adaptation-Evolution, Université de Nice-Sophia Antipolis, 06108 Nice Cedex 2, France; Université de Nice-Sophia Antipolis Nice UMR 7138, Systématique-Adaptation-Evolution, 06108 Nice Cedex 2, France; Department of Zoology, Oklahoma State University, Stillwater, OK 74078 USA; Department of Biological Sciences, University of Alberta, Edmonton, AB T6G 2E9 Canada; Bamfield Marine Sciences Centre, Bamfield, BC V0R 1B0 Canada; Centre Scientifique de Monaco (CSM), ᅟ, Monaco

## Abstract

**Background:**

The Ras/Raf/MEK/ERK signaling pathway is involved in essential cell processes and it is abnormally activated in ~30 % of cancers and cognitive disorders. Two ERK isoforms have been described, ERK1 and ERK2; ERK2 being regarded by many as essential due to the embryonic lethality of ERK2 knock-out mice, whereas mice lacking ERK1 are viable and fertile. The controversial question of why we have two ERKs and whether they have differential functions or display functional redundancy has not yet been resolved.

**Results:**

To investigate this question we used a novel approach based on comparing the evolution of ERK isoforms’ sequences *and* protein expression across vertebrates. We gathered and cloned erk1 and erk2 coding sequences *and* we examined protein expression of isoforms in brain extracts in all major clades of vertebrate evolution. For the first time, we measured each isoforms’ relative protein level in phylogenetically distant animals using anti-phospho antibodies targeting active ERKs. We demonstrate that squamates (lizards, snakes and geckos), despite having both genes, do not express ERK2 protein whereas other tetrapods either do not express ERK1 protein or have lost the *erk1* gene. To demonstrate the unexpected squamates’ lack of ERK2 expression, we targeted each ERK isoform in lizard primary fibroblasts by specific siRNA-mediated knockdown. We also found that undetectable expression of ERK2 in lizard is compensated by a greater strength of lizard’s *erk1* promoter. Finally, phylogenetic analysis revealed that ERK1 amino acids sequences evolve faster than ERK2’s likely due to genomic factors, including a large difference in gene size, rather than from functional differences since amino acids essential for function are kept invariant.

**Conclusions:**

ERK isoforms appeared by a single gene duplication at the onset of vertebrate evolution at least 400 Mya. Our results demonstrate that tetrapods can live by expressing either one or both ERK isoforms, supporting the notion that ERK1/2 act interchangeably. Substrate recognition sites and catalytic cleft are nearly invariant in all vertebrate ERKs further suggesting functional redundancy. We suggest that future ERK research should shift towards understanding the role and regulation of total ERK quantity, especially in light of newly described *erk2* gene amplification identified in tumors.

**Electronic supplementary material:**

The online version of this article (doi:10.1186/s12862-015-0450-x) contains supplementary material, which is available to authorized users.

## Background

ERKs are the effector kinases of the Ras/Raf/MEK/ERK signaling pathway involved in multiple essential cell processes such as proliferation [1], differentiation [2], survival and memory formation [3, 4]. Abnormal activation of this cascade leads to pathologies such as cancer [5] or cognitive impairments [6]. Since the discovery of two ERK isoforms in mammals, ERK1 (MAPK3) and ERK2 (MAPK1) in 1991 [7], numerous researchers have strived to understand their respective roles. While some cancers have been associated with isoforms of ERK cascade members such as B-Raf for melanoma [8], the putative differential involvement of either ERK isoform in cancer or any disease remains unknown.

ERK1 and ERK2 are expressed ubiquitously in mammals where both display the same kinase specific activity *in vitro* [9, 10] and share a highly similar 3D structure (Additional file 1C). Furthermore in mammals, both translocate to the nucleus upon stimulation by cell surface receptors [11] and while they do share at least 284 interactors, no isoform-specific substrates have been identified [12]. Indeed, ERK1 and ERK2 share 22 out of 23 amino acids that have been demonstrated to directly interact with substrates [13, 14], the sole difference being a conservative substitution: leucine^155ERK2^ into isoleucine^175ERK1^ (Additional file 1A).

ERK2 is regarded by many as essential due to the embryonic lethality of ERK2 knock-out mice [15–17], whereas mice lacking ERK1 are viable and fertile suggesting a dispensable role of ERK1 [18]. Similarly, some studies based on siRNA mediated-invalidations suggest specific functions [19–21]. However, targeted *erk1* and/or *erk2* gene disruption in mice organs can evoke redundancy [22]. In mouse fibroblasts we showed that solely si-RNA mediated ERK2 knock-down reduced cell proliferation by itself, however when ERK2 levels were clamped down, ERK1 knock-down became effective at reducing cell proliferation. Hence, we and others have hypothesized that the apparent dominant role of ERK2 is only due to its higher expression rather than functional differences [10, 23]. To date, the controversial question of ERKs differential function versus redundancy has not been successfully addressed.

Here we show by a combined approach based on ERK1/2 sequence evolution and ERK1/2 protein expression across vertebrates, that while some tetrapods express both ERK1 and ERK2 proteins, others have lost the *erk1* gene and others express either only ERK1 or only ERK2 at detectable levels despite having both genes. Hence our results strongly suggest that ERK1/2 can act interchangeably, a conclusion strengthened by the observation that amino-acids required for function are invariant in ERK1 and ERK2.

## Results

**Fig. 1:**
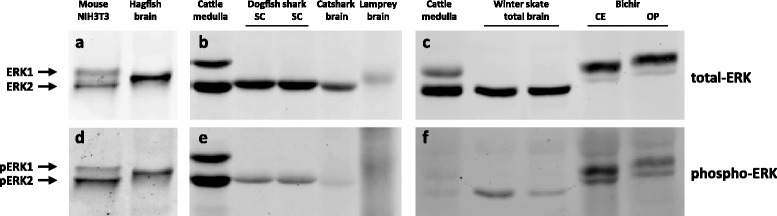
ERK1 and ERK2 protein appear first in bony vertebrates (Bichir). Western-blot analysis of samples from brain, spinal cord or cultured cells: immunoblots were incubated with polyclonal anti-ERK antibody (ERK1 and ERK2; panels **a**, **b**, **c**) or anti-phosphoERK antibody (pERK1 and pERK2; panels **d**, **e**, **f**). Taxonomic names are listed in Additional file 7. Predicted MW sizes of vertebrate ERKs range from 41.7 kDa for lamprey’s ERK to 44.1 for zebrafish’s ERK1. SC: spinal cord; CE: cerebellum; OP: optic lobe. Positions of ERK1, ERK2, phospho-ERK1 (pERK1) and phospho-ERK2 (pERK2) are indicated on the sides

All mammals tested to date express both ERK1 and ERK2 from eutherians (mouse, rat, rabbit, cattle) to the marsupial opossum (Additional file 2D and [10]). To search clues for specific functions or functional redundancy of ERK isoforms, we studied protein expression of ERK isoforms in parallel with the evolution of their sequences in all key vertebrate clades. We chose to study protein expression in brains to allow comparison across very divergent species and gain high sensitivity since in mammals, this organ has the greatest level of ERK expression. [7]. To ensure detection specificity we performed western blots incubated with anti-ERK and anti-phospho-ERK antibodies. In vertebrates, ERK1 and ERK2 are highly conserved, thus allowing various antibodies to recognize evolutionarily distant ERKs. Furthermore, the epitope recognizing doubly-phosphorylated ERK is conserved from sea anemones to humans, contributing to unambiguous protein identification (Additional file 3A). As a proof of principle for this approach, we can readily detect a single ERK protein with three distinct anti-phospho ERK antibodies and one antibody targeting total-ERK in hagfish (*Eptatretus stoutii*), the most evolutionary divergent vertebrate specie compared to mammals (Fig. 1a,d and Additional file 3B).

### One ERK is expressed in agnathans and chondrichthyes

**Fig. 2:**
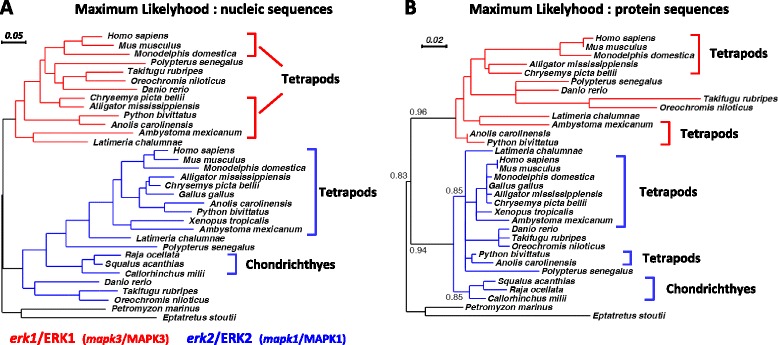
Evolution of vertebrate ERK sequences. **a** Maximium-likelihood phylogenetic analysis of *erk* nucleotide and (**b**) derived ERK amino-acid sequences, at key nodes of vertebrate evolution. *erk1*/ERK1 and *erk2*/ERK2 phylogenies are indicated by red and blue branches respectively. Key taxonomic clades for this manuscript (tetrapods and Chondrichthyes) are indicated by brackets. Accession numbers and common names are listed in Additional file 7. Branch length is indicative of degree of sequence divergences. The order of species in the derived trees is noted to vary slightly between different topological methods and whether it is calculated using protein or nucleotide sequences. Given that all vertebrate ERK sequences are highly conserved, minor variations in tree placement can be expected

Lamprey (*Petromyzon marinus)* belongs to the same Agnathan clade as hagfish and possesses only a single *erk* gene within its genome [24] and we detected only one ERK in lamprey’s brain extracts (Fig. 1b,e). Phylogenetic analysis of hagfish and lamprey ERKs sequences highlight the similarity of the single ERK from these species (Fig. 2).

The next major clade in the vertebrate lineage, cartilaginous fishes (class Chondrichthyes) are constituted of three sub-clades: chimeras, sharks and rays. Among chimeras, the genome of the elephant shark (*Callorhinchus milii*) is sequenced and has only a single *erk* [25]. To extend this result to the two other classes of chondrichthyes we first analyzed ERK expression in brains. We detected a sole ERK isoform in the brain of winter skate (*Raja ocellata*), medulla of the small-spotted catshark (*Scyliorhinus canicula*) and spinal cord of the spiny dogfish (*Squalus acanthias*, Fig. 1). Using degenerated primers targeting teleost *erk1* and *erk2*, we also amplified and cloned a single *er*k from winter skate (ray) and spiny dogfish (shark), both of which share a high sequence identity (87–89 % at nucleotide level) to elephant shark *erk*. We conclude that members of the three clades of Chondrichthyes express a single ERK.

The single ERK of Chondrichthyes is likely to be ERK2, firstly because its sequence segregates within ERK2 group by three independent topological methods (Fig. 2 and Additional file 5), and the bootstrap values of the branches of Fig. 2 that place Chondrichthyes ERK into the ERK2 group support this finding. Secondly, the size of the *erk* gene from elephant shark is large (55 kb), similar to the large size of teleost and mammal *erk2* genes and opposite of the extremely compact *erk1* genes present in vertebrates as it will be described at the end of the result section (Fig. 6).

### ERK1 and ERK2 from all bony vertebrates arose from a single duplication event

All teleosts genomes sequenced so far (*e.g. Danio rerio and Fugu rubripes*) possess two ERK genes; and we readily detect two ERK proteins from brain extracts of two representatives of the same teleost clade: *Dicentrarchus labrax* and *Hemichromis bimaculatus* (Additional file 2A).

Bichirs are extant representatives of the most ancient branch of ray-finned fishes, diverging from the rest of Actinopterygii prior to the whole genome duplication specific of teleosts [26]. Therefore ERK isoform identification in bichir is critical for determining ERK1 and ERK2 emergence in bony fish. We analyzed brain extracts from bichir (*Polypterus senegalus)* and detected two ERK isoforms (Fig. 1A and Additional file 2A) and we further cloned two *erk* genes from *P. senegalus*. Phylogenetic analysis of bichir ERK isoforms revealed that they already fully segregate at gene and protein levels as ERK1 and ERK2 as classified in teleosts and tetrapods (Fig. 2 and Additional file 5). This demonstrates duplicate ERK1/2 emergence at least 400 Mya prior to the divergence of the bichirs from primitive bony vertebrates [26]. Furthermore, in human and zebrafish genomes *ypel3* gene is located downstream of *erk1/mapk3* gene in the same orientation than *erk1*; and *ypel1* is located downstream of *erk2/mapk1* in the same orientation than *erk2*; *ypel1* and *ypel3* being also isoforms therefore the synteny of isoforms is conserved from fish to mammals between these two loci, which is further indicative of a single duplication event that generated ERK1 and ERK2 in vertebrates.

### *erk1* gene has been lost independently in two tetrapod clades

**Fig. 3:**
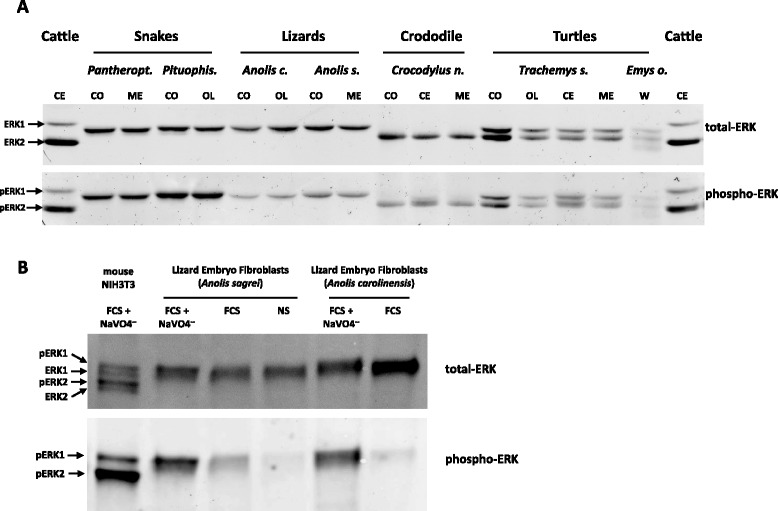
ERK2 is not detected in snakes and lizards. **a**, **b** Western-blot analysis performed as in Fig. 1 incubated with a polyclonal anti-ERK antibody (upper panels) or anti-phosphoERK antibody (lower panels). **a** brain extracts from snakes, lizards, crocodile and turtles (full taxonomic names in Additional file 7). Control brain extracts from cattle. CO: cortex, CE: cerebellum, ME: medulla, OL: olfactory bulb, PI: pineal gland, W: whole brain. Note that degraded bands are observed in turtle *E. orbicularis* likely due postmortem sampling. **b** extracts from lizard embryo fibroblasts were stimulated or not (NS) one hour in presence of FCS alone (FCS) or FCS + phosphatase inhibitor NaVO4^—^(FCS + NaVO4^—^) to activate ERKs

Western blot studies and gene sequencing revealed that several extant members of the three tetrapod clades express both ERK1 and ERK2: turtles for the sauropsid clade Fig. 3a), axolotl for the amphibian clade (Additional file 2B) and mouse for the mammalian clade (Additional file 2D and [10]). However, only one ERK was detected in Western blots of protein extracts from African clawed frog (Additional file 2B) and chicken (Additional file 2C). The search of exons in fully sequenced genomes combined with the screening of EST databases reveals that the *erk*1 gene is absent from the genomes of *Xenopus tropicalis* (amphibian), chicken (*Gallus gallus*, a neo-gnathae) [27] and 4 other recently published genomes of neo-gnathae (Ensembl release 75). Furthermore, ERK1 protein expression is not detectable in the brain of the paleo-gnathae nandu (*Rhea. americana*, Additional file 2C), and no *erk1* gene was identified in the genome of the paleo-gnathae ostrich (*Struthio camelus*). These observations suggest that *erk*1 has been lost in all bird lineages and in some amphibians. As summarized in Table 1, only *erk1* gene has been lost in some tetrapods and all chondrichthyes.

**Table 1:** Birds, frogs and cartilaginous fishes lack *erk1* gene in their genome

					Genes present		Proteins detected by western blot	
					erk1	erk2	ERK1	ERK2
Mammalia	Eutheria		Mouse	*Mus musculus*	✓	✓	✓	✓
			Cattle	*Bos taurus*	✓	✓	✓	✓
			Chinese hamster	*Cricetulus griseus*	✓	✓	✓	✓
			European rabbit	*Oryctolagus cuniculus*	✓	✓	✓	✓
	Metatheria		Opossum	*Monodelphis Domestica*	✓	✓	✓	✓
	Monotremata		Platypus	*Ornithorhynchus anatinus*	✓	✓	nd	nd
Sauropsida	Archosauria	Aves	Chicken	*Gallus gallus*	**o**	✓		✓
			Greater Rhea	*Rhea americana*	**o**	✓		✓
		Crocodylia	Nile crocodile	*Crocodylus niloticus*	✓	✓		✓
	Squamata		Garter snake	*Thamnophis elegans*	✓	✓	✓	
			Corn snake	*Pantherophis guttatus*	✓	✓	✓	
			Bull snake	*Pituophis catenifer sayi*	✓	✓	✓	
			Green anole	*Anolis carolinensis*	✓	✓	✓	
			Brown anole	*Anolis sagrei*	✓	✓	✓	
			Common gecko	*Tarentola mauritanica*	✓	✓	✓	
	Testudines		Red-eared slider	*Trachemys scripta elegans*	✓	✓	✓	✓
			European pond turtle	*Emys orbicularis*	✓	✓	✓	✓
Amphibia		Anura	African clawed frog	*Xenopus laevi*	**o**	✓		✓
		Caudata	Axolotl	*Ambiostoma mexicanum*	✓	✓	✓	✓
Presence in fishes								
Euteleostomi	Sarcopterygii		Coelacanth	*Latimeria chalumnae*	✓	✓	nd	nd
	Actinopterygii	Teleosts	European sea bass	*Dicentrarchus labrax*	✓	✓	✓	✓
			African jewelfish	*Hemichromis bimaculatus*	✓	✓	✓	✓
		Polypteridae	Bichir	*Polypterus senegalus*	✓	✓	✓	✓
Chondrichthyes			Spiny dogfish	*Squalus acanthias*	**o**	✓		✓
			Smaller spotted catshark	*Scyliorhinus canicula*	**o**	✓		✓
			Winter skate	*Raja ocellata*	**o**	✓		✓

Genomes and EST databases were scrutinized for the presence of *erk1/2* exons, if gene is present: ✓ if gene is absent: O

Presence of proteins readily detected on Western Blots in this work is indicated: ✓

*nd* not determined

### In reptiles either one or both ERKs are expressed

**Fig. 4:**
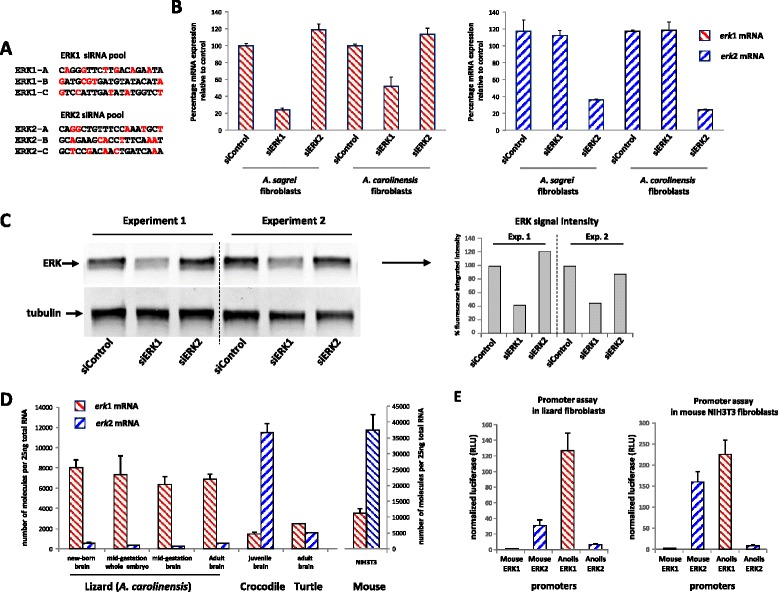
Only ERK1 protein and erk1 mRNA are expressed significantly in lizards. **a** siRNA sequences targeting anole lizard (*A. carolinensis*) erk1 mRNA (ERK1-A, ERK1-B and ERK1-C) and anole lizard erk2 mRNA (ERK2-A, ERK2-B and ERK2-C). In red, the nucleotides that differ when both erk1 and erk2 isoforms are aligned. **b**, **c** siRNA pools targeting erk1 or erk2 or control-siRNA were transfected three days prior to mRNA harvesting. **b** Absolute quantification of erk1 mRNA (left panel) or erk2 mRNA (right panel) after siRNA transfection, in *A. sagrei* and *A. carolinensis* lizard embryo fibroblasts (LEFs). erk1 or erk2 mRNA quantities were determined relatively to quantities of linearized plasmids harboring either *A. carolinensis* erk1 or erk2 cDNAs as described in materials and methods. To ease comparison between erk1 and erk2, data are expressed as percentage of siControl quantities. Bars represent mean +/− s.d. *n* = 4. **c** Western-blot analysis of samples from two independent transfections loaded on the same gel (Experiment 1 and 2) (insert) Fluorescence quantification of western blot. **d** Number of erk1 and erk2 mRNA molecules per 25 ng of total RNA from reptile samples: lizard (*A. carolinensis*), Crocodile (*C. niloticus*), turtle (*T. scripta elegans*) and mouse NIH3T3 cells. Bars represent means +/− s.d. *n* = 3 (**e**) Firefly luciferase activity driven by mouse and anole lizard ERK promoters (1 kb upstream from start ATG). Normalization by co-transfection of a plasmid expressing *Renilla* luciferase driven by the thymidine kinase promoter. Transfection was performed in *A. carolinensis* fibroblasts (left panel) or mouse NIH3T3 fibroblasts (right panel). Bars represent means +/− s.d. *n* = 4

Both ERK proteins are expressed in turtle brain, however crocodiles appear to express only ERK2 protein in the same organ (Fig. 3a). By contrast, we could only detect ERK1 protein in snakes and lizards (Fig. 3a). Side-by-side loading of cerebellum extracts from lizard, crocodile and turtle confirm these observations (Additional file 6A). Furthermore, an apparent lack of ERK2 was observed in brain extracts from all squamate species tested: 4 lizards, 4 snakes and one gecko (Additional file 6B and Table 1). The two reptile genomes sequenced so far possess *erk*1 and *erk*2 (lizard *A. carolinensis* and snake *P. bivittatus*); therefore to confirm the surprising absence of ERK2 protein in the model animal for this clade, *A. carolinensis* [28], we first confirmed the absence of ERK2 expression in an array of *A. carolinensis* adult tissues (Additional file 6C). We then derived primary lizard embryo fibroblasts (LEFs) from mid-gestation embryos. Even when ERK was hyper-stimulated by serum in presence of orthovanadate to increase detection with anti-phospho ERK antibodies, only a single ERK1-sized band was observed (Fig. 3b). To unambiguously assign protein identity and remove the possibility that two ERK proteins are present and co-migrate, LEFs were transfected with pools of three independent siRNA sequences targeting *erk*1 or *erk*2. At minimum, each siRNA displays 5 differences with the non-targeted isoform to ensure isoform-specificity (Fig. 4a). Efficiency and isoform specificity of each siRNA pool was verified by measuring *erk1/2* mRNA levels post-transfection (Fig. 4b). Three days post-transfection, the expression level of *A. carolinensis* ERK protein was consistently lowered by siRNA targeting *erk*1 whereas siRNA targeting *erk*2 had no effect (40–60 % reduction by erk1-siRNA in 5 experiments, Fig. 4c). Therefore, we suggest with a strong degree of evidence that ERK1 is the only protein isoform detected in anole lizard, and more generally in squamates.

This raises the interesting question of why ERK2 is not detected in squamates. Quantitative measurements of *erk*1/2 mRNA expression reveal that *erk*1 mRNA expression is 12- to 24-fold higher than *erk*2 in *A. carolinensis* tissues (mid-gestation embryo or brain, neo-natal brain and adult brain (Fig. 4d). This markedly contrasts with juvenile crocodile brain where *erk*2 mRNA expression is 8-fold greater than *erk*1 and with mouse NIH3T3 cells where *erk*2 mRNA expression is 3- to 4-fold greater than *erk*1. Conversely, in adult turtle brain, *erk*1 and *erk*2 mRNAs are expressed at similar levels (Fig. 4d). In all these animals, protein expression of ERK isoforms correlates well with mRNA expression therefore lizards express undetectable ERK2 protein due to minimal *erk*2 mRNA expression.

We then compared opposite mammalian versus squamate ERKs expression by cloning mouse and anole lizard *erk*1 and *erk*2 promoters upstream of the luciferase reporter gene (1 kb upstream initiating codon). In cells from these organisms, transient transfection revealed that mouse *erk*1 promoter is much weaker than mouse *erk*2 promoter; conversely *A. carolinensis erk*1 promoter is markedly stronger than *A. carolinensis erk*2 promoter (nearly 20-fold more in lizard fibroblasts, Fig. 4e). Hence, the low expression of ERK1 in mouse and low expression of ERK2 in anole lizard seem to originate from the weakness of their respective promoters. The difference of strength between mouse *erk* promoters is larger than the protein ratio observed in the same cells; therefore further research is needed to understand the individual contribution of promoters, RNA regulation and protein stability to establish the final ERK1/ERK2 protein ratio.

### ERK1 evolve faster, but amino-acids required for kinase function are invariant in ERK1 and ERK2

**Fig. 5:**
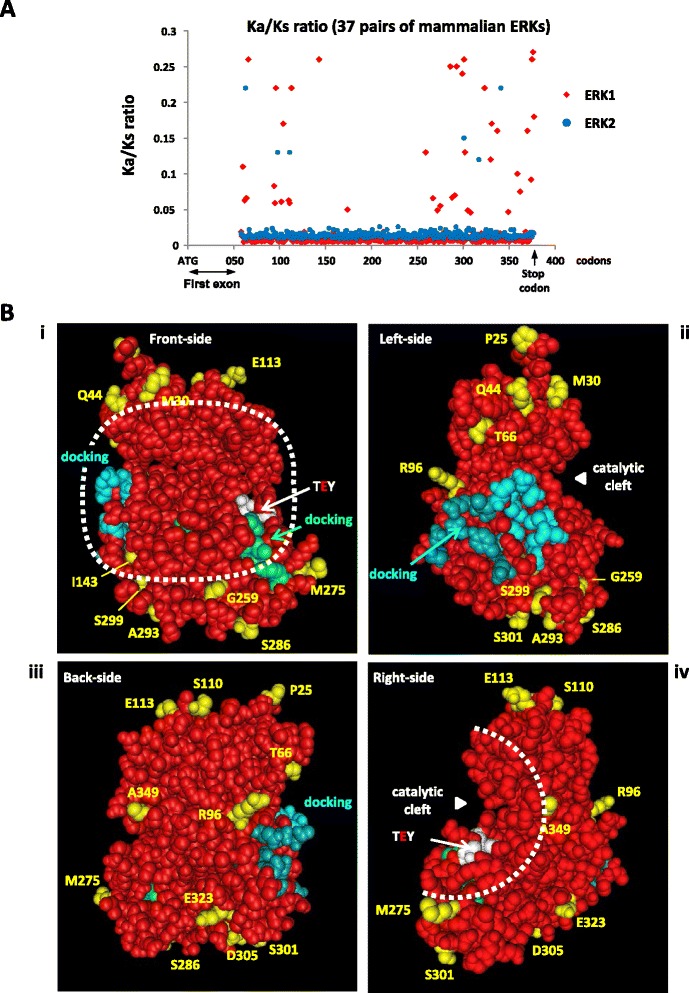
Only amino-acid neutral for function diverge among mammalian ERKs. **a** Ratio of non-synonymous rate (Ka) over synonymous rate (Ks) for all available *erk1* and *erk2* pairs of mammalian gene sequences (37 *pairs*). Multiple sequence alignment was generated and Ka/Ks values were then computed at each codon. Abscissa: distance from start ATG codon. The 5′ end of the sequences (first exon) could not be analyzed because some sequences were incomplete. **b** Still images were generated for 3D PDB structure 4QTB (Human ERK1) displaying the following highlighted positions: amino-acids interacting with substrates forming the DEJL(KIM) motif are colored in blue, light blue for docking groove and darker blue for acidic patch; amino-acids interacting with substrates via the DEF domain (FXFP) are colored in green; the threonine and tyrosine phosphorylated upon activation by MEK are colored in white and the “face of the kinase” is circled by a dashed white line. Amino-acids that diverge among at least 5 % of 49 mammalian ERK1s are colored in yellow (17 amino-acids). i) front-side of the kinase where ATP-transfer occurs in the catalytic cleft between the two lobes ii) left-side of the kinase iii) back-side of the kinase iv) right-side of the kinase

In order to compare erks nucleotide and ERKs protein sequence evolution, we generated phylogenetic trees. We found that *erk* nucleic sequences displayed similar branch lengths for *erk1* and *erk2*; however, at the protein level, the branch lengths were markedly longer for ERK1 proteins than for ERK2 proteins (Fig. 2 and Additional file 5) indicating that the rate of evolution at the gene level is similar between *erk*1 and *erk*2 but the rate of protein evolution is higher for ERK1 than ERK2. This trend was confirmed by showing in Table 2 that the synonymous mutation rates (Ks) of vertebrate ERK1 and ERK2 sequences are nearly identical (0.640 and 0.717 respectively) whereas the non-synonymous rate of mutation (Ka) is more elevated for ERK1 (0.083) than ERK2 (0.033). Indeed, ERK2 was shown to display the lowest evolution rate of vertebrate MAP Kinases [29]. To scrutinize if differences of evolutionary rates between ERK1 and ERK2 is ongoing, we restricted our analysis on mammalian *erk*1/2 pairs from the same organism, which also ensures that they underwent the same evolutionary pressure. We show that the Ka/Ks ratio is extremely low along the mammalian ERK2 sequence, which is typically the case during purifying selection (only neutral mutations are kept, Fig. 5a). ERK1 is also under strong purifying selection along the sequence but not as much as ERK2, confirming that evolution of mammalian ERK1 sequences is still greater than ERK2 sequences (Fig. 5a). This is confirmed by the global calculation of Ka and Ks ratios for 94 mammalian ERKs, which reveal that ERK1 Ka/Ks ratio is 4.45 fold higher than ERK2’s (Table 2). By calculating Shannon entropy along the sequences of all available pairs of full-length mammalian ERK1/2 we show that nucleotides divergences are widespread along the sequences of both erks (Additional file 5F), whereas mammalian ERK1 proteins sequences diverge markedly more than ERK2, especially at the N- and C-terminal ends away from the kinase core (Additional file 5E). On the 3D structure of human ERK1, the amino acids that diverge from consensus in at least 5 % of 49 mammalian ERK1s were highlighted. Apart from Ile^143^, all amino-acids are localized at the surface of the molecule and Ileu^143^ is replaced only with a valine which is a conservative change (Fig. 5b). More importantly, all these divergent amino-acids are localized on the back of the kinase, distally from the catalytic cleft of the kinase and docking domains of ERK for substrates and interactors (thereafter named the face of the kinase, Fig. 5b). Hence this faster evolution of ERK1 occurs at positions neutral for function.

**Table 2:** ERK1s protein sequences evolve faster than ERK2s’, not their nucleotide sequences

	Vertebrate ERKs		Mammalian ERKs	
	ERK1	ERK2	ERK1	ERK2
Number of codons analysed	298	298	324	322
Nucleotide substitution rate	0,221	0,210	0,091	0,080
Amino acid substitution rate	0,077	0,031	0,020	0,004
Nonsynonymous substitution rate	0,052	0,025	0,010	0,003
Synonymous substitution rate	0,640	0,717	0,321	0,322
Nonsynonymous/synonymous substitution rate	0,083	0,033	0,029	0,007
(Ka/Ks Ratio of ERK1) / (Ka/Ks Ratio of ERK2)	2,54		4,45	

The synonymous substitution rates (Ks) and the non-synonymous substitution rates (Ka) of vertebrate ERKs and mammalian ERKs were calculated in MEGA6 as described in Methods

## Discussion

The present study reveals that ERK1 and ERK2 identities are well separated in tetrapods, indicative that they arose from a single duplication event. The synteny between ERK isoforms with YPEL isoforms is conserved at least in human and zebrafish indicating that a segment of chromosome has duplicated. Furthermore, genome segments encompassing human ERK1 and human ERK2 display patterns of synteny with a single amphioxus linkage group [30]. Taken together, these elements are indicative that ERK1 and ERK2 likely originated from the vertebrate-specific whole genome duplications (WGDs). Prior to bony fish, all vertebrates express only one ERK. Considering that two rounds of WGD occurred prior to divergence of gnathostome lineages [24], we conclude that paralogous ERK duplicates were lost in Chondrichthyes. The resolution of phylogenetic analysis in these deep vertebrate branches does not allow to classify the single *erk* of Agnathans as ERK1 or ERK2, however extant Chondrichthyes seem to express the ERK2 isoform, a conclusion confirmed by the large size of the *erk* gene of elephant shark fish, a unique characteristic of *erk2* genes in all bony vertebrates.

It has been hypothesized that gene duplicates are usually lost during evolution after a WGD if no new function appear among paralogs [31]. The predominant loss of ERK1 protein expression from cartilaginous fishes to birds, and lack of detectable ERK1 expression in crocodiles mimics the lack of major phenotype of mice invalidated for the *erk1* gene. This could suggest that ERK2 carries ancestral and essential ERK function and therefore its expression is required in vertebrates. At the protein level, however the results presented here within the reptile clade shatter this hypothesis since we demonstrate that ERK2 is not detected in squamates. The identities of lizard ERK isoforms are kept since the sequence of ERK2 from the lizard *A. carolinensis* is very similar to the sequence of mouse ERK2 (97 % identity, Additional file 1B) and amino acid position that diverge between lizard ERK2 and human ERK2 are all localized distally from the face of the kinase (Additional file 1D). Similarly lizard ERK1 sequence is 91 % identical to that of mouse ERK1 and all divergent amino acids are located distally from the face of the kinase, except the conservative substitutions serine^135-humanERK1^ for threonine^132-anolisERK1^ and glutamic-acid^194-humanERK1^ for aspartic-acid^191-anolisERK1^ (Additional file 1E). Hence, in squamates, ERK1 and ERK2 have kept their identity but only ERK1 is significantly expressed. Though undetectable relatively to ERK1 by our methods, squamates’ ERK2 is certainly expressed since it is still under selective pressure to remain free of nonsense mutations. Similarly, in crocodiles ERK1 protein is undetectable relative to ERK2 and its sequence remains fully functional however, it is unlikely to play a specific role since in the same clade of Archosaurians since birds have lost *erk1* altogether. The extinction of ERK1 protein expression in crocodiles may even be a premise to the loss of *erk*1 gene in birds since birds diverged later than crocodiles from their common ancestor. We propose that both ERKs contribute to reach a global threshold concentration which maintains selective pressure on isoforms’ functionality. Even in adults, a minimum threshold of ERK is required to sustain vertebrate life (as demonstrated whereby partial loss of ERK upon gene invalidation mediated by CRE-recombinase induction, leads to rapid death by multiple organ failures) [32]. Considering that in vertebrates expressing both functional ERK proteins, ERK quantity can be overwhelmingly provided by ERK1 or by ERK2, our results suggest that ERK1 and ERK2 are functionally redundant. In mammals, two gene loci may increase the ability to regulate exquisitely the quantity of ERK during all life stages. During the revision of this manuscript, Meloche and co-workers showed that mice completely lacking ERK2 expression were rescued only when the ERK1 was overexpressed, suggesting a full redundancy of ERK1/2 proteins during development up to normal reproduction [33].

**Fig. 6:**
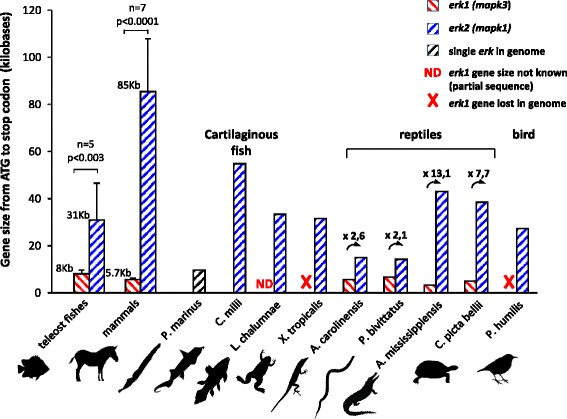
In vertebrates, *erk2* genes are larger than *erk1* genes. Genomes from vertebrates were screened for the presence of the full length sequences of *erk* genes, and then the size from ATG to stop codon was calculated in kilobases. In teleosts and mammals, animals for which both *erk1* and *erk2* genes sequences are available were preferred, only one pair of mammalian *erks* is not from the same animal. Full *erk1* gene sequence of coelacanth (*L. chalumnae*) is not available. For teleosts, the *erk* genes of *T. fubripes* and *T. nigroviridis* were not taken into account due to their known extreme genome compactness that would skew the size distribution (smallests vertebrate genomes) their *erk1* gene size being 5 and 3.9 kb respectively and their *erk2* gene size being 9.7 and 10 kb respectively). For teleosts and mammals, the average sizes of the genes are written on the upper left side of the graphical bars, and the p-value that evaluates the statistical significance of the difference between *erk1* and *erk2* gene sizes is indicated. For reptiles, the fold difference between the sizes of *erk1* genes and *erk2* genes is indicated on top of the bars with an arrow. Silhouettes are from http://phylopic.org/

*erk1* genes are lost more often than *erk2’s* in vertebrates and mammalian ERK1s evolve faster than ERK2s’ which raises the possibility that these two observations are linked. In multicellular organisms three independent criteria determine the evolution rate of proteins [34]: gene essentiality, expression level [35] and gene compactness [36]. The faster evolution rate of ERK1 cannot be attributed to differences in gene essentiality since so far no differences in functions have been demonstrated between ERK1 and ERK2 and because tetrapods express either one or both isoforms. Despite being a minor determinant of protein evolution in multicellular organisms, low protein expression of ERK1 could play a role in the faster evolution rate of ERK1 since ERK1 is expressed usually at lower levels than ERK2 (in brain from marsupial to mouse Additional file 2D, and in nearly all mouse tissues tested Additional file 4). More strikingly, the difference in gene compactness between mammalian *erks* is tremendous, *erk1* gene being in average 15 fold smaller than *erk2* (Fig. 6). Several authors have correlated small gene size with faster evolution rate, by decreasing the probability of recombination between exons to purify mutations (Hill and Robertson interference hypothesis [36, 37]. Interestingly, among reptiles the gene size differences between *erk1* and *erk2* are minimal for squamates (about 2 fold) compared to crocodile (13 fold) and turtle (7 fold, Fig. 6). Furthermore squamates’ ERK1 and ERK2 proteins seem to evolve at similar rate since there is no branch length bias in the phylogenetic analysis, unlike for all other bony vertebrates (Fig. 2 and Additional file 5B). Hence there seem to be a correlation between lower differences of *erk* gene sizes in squamate and lower difference between ERKs protein evolution rates. The availability of many squamate genomes is needed to confirm this trend. Hence, except in squamates that present *erk* genes of similar sizes, a larger gene size may explain the ERK2 slower protein evolution, and specific regulation(s) of ERK2 expression derived from the larger gene size, may explain the observed ERK2 prevalence in vertebrates.

## Conclusion

Our study suggests that ERK1 and ERK2 are functionally redundant in tetrapods since either one or both isoforms are expressed in animals from the same sauropsidian clade (reptiles and birds). ERK1 proteins evolve faster than ERK2 proteins, even when the study was restricted to the mammalian clade; however amino acids diverge only at position neutral for function hence we propose that the origin of this difference originates from genomic factors such as the large gene size difference, but not from functional differences. It remains to be determined whether the large gene size differences may also explain why *erk1* genes are lost more readily than erk2’s. When evaluating ERK1/2 invalidations, observed phenotypes should now be linked to the quantitative amount of total-ERK reduction, not to functional differences among isoforms. ERKs being the most expressed proteins of this signaling cascade, future research should aim at understanding the regulation and role of total ERK quantity in cells.

## Methods

### Cell culture and reagents

Lizard embryo-fibroblasts preparation was adapted from [38]: Eggs of mid-gestation embryos of A. *carolinensis* or A. *sagrei* were sterilized by three washes with 70 % ethanol and cut open by scalpel. Embryos were washed in sterile PBS, and sectioned into approximately 1 mm^3^ cubes to be incubated at 37 °C in DMEM medium complemented with 0.25 % trypsin (Gibco #15090046), 2 mg/ml collagenase (Sigma # C2674), 2 mg/ml DNAse (Sigma DN25) and 5 mM MgCl_2_. Gentle pipetting was applied to aid in cell dispersal. After 30 min treatment, the suspended cells were collected and centrifuged at 16.1x *g* for 5 min while chunks of tissues were incubated for an additional 30 min with fresh medium prior to centrifugation.

Supernatants were drawn off and cellular pellets were re-suspended in DMEM medium (GIBCO 61965–026) supplemented with 5 % chicken serum (sigma # C5405), 10 % FCS (Dominique Dutsher-South American serum), penicillin and streptomycin then plated on 6-well plates (Falcon #353046). Some cells were grown in the same medium supplemented with Nutrient mixture F (Sigma # N6013) with no detectable advantages. Plated cells were incubated at 37 °C in 5 % CO_2_ for 1 week. Cells were then cultivated in DMEM supplemented with 8 % FCS only. Cell-passage occurred by trypsinizing confluent cells following PBS washes, and plating cells with 3-fold dilution. Cells remain viable for less than 10 cell-passages. Cells observed to be rapidly dividing were frozen in aliquots in presence of 10 % DMSO, DMEM and 50 % serum to be studied later.

Mouse NIH 3 T3 cells, Human A375 and Chinese Hamster CCL39 cells were obtained from the AmericanType Culture Collection.

### siRNA transfection

6 μl of Ribocellin (BioCellChalenge; France) was mixed with siRNAs in 100 μl PBS (each siRNA is transfected at a final concentration of 60nM; 180 nM for the control). This mix was added to lizard fibroblasts cultivated in 1 ml of serum-containing medium in 35 mm plates. Cells were cultivated in presence of siRNA for 3 days. Each extract loaded on gel represents harvesting from two 35 mm plates. siRNA were synthetized and annealed by Eurogentec (Belgium), sequences targeting *A. carolinensis* are presented in Fig. 4a and the Si-control (si-luciferase) sequence was: CGTACGCGGAATACTTCGA.

### Immunoblotting

Brains (stored at −80 °C) were dissected, lysed in laemmli sample buffer, sonicated and then boiled at 95 °C for 8 min. Protein concentrations of brain extracts and cell lysates were measured by the bicinchoninic acid method (Pierce). Tissue samples from *A. carolinenis* where incubated in RNA later for shipment, chunks were isolated by centrifugation and frozen in liquid nitrogen prior being smashed into powder. Frozen powder was dissolved into triton lysis buffer containing proteases and phosphatases inhibitor for 30 min, and then centrifuged at 20000 g for 30 min. Supernatant was diluted 2 folds into 3X laemmli sample buffer prior boiling. Proteins were separated by sodium dodecyl sulfate (SDS)-polyacrylamide gel electrophoresis (10 % acrylamide-bis acrylamide [29:1] gels) loaded with 15 μg to 40 μg of protein per lane depending on gel size. Proteins were transferred onto nitrocellulose membranes: Hybond RPN303D from Amersham. Membranes were blocked by incubating with 2.5 % BSA in PBS containing 0.12 % gelatin, 0.1 % casein. Antibodies were incubated in PBS containing 0.1 % gelatin and 0.08 % casein. Phosphorylated ERK1/2 was detected with the mouse monoclonal anti-phospho-ERK1/2 antibody (Sigma M8159), identical results were obtained with the rabbit monoclonal anti-phospho ERK (Cell Signaling #4370). Total ERK was detected with the rabbit monoclonal anti-ERK (Cell Signaling #4695), or the rabbit anti-rat ERK1 (1:4,000; Fisher 1019–9152) or mouse monoclonal ERK2 (1:5,000; BD Pharmingen #610103) plus mouse monoclonal ERK1 (1:1,000, BD Pharmingen #554100). Secondary antibodies were IR dyes anti-rabbit 800CW (1:1000) and anti-mouse 680RD (1:1000) from Li-cor.

### DNA sequences

*erk* coding sequences were gathered from genomic sequencing by searching the Ensembl database or by exon search by BLAST in NCBI database or ambystoma.org database. Hagfish *erk* sequence was obtained from transcriptomic sequencing. For animals with unknown *erk* sequences, tissues fragments were dissolved into 1 ml TRIzol reagent (# 15596–018 Life Technologies), aqueous phase containing RNA was separated by adding 20 % chloroform, then RNA isolation was performed with aqueous solution with RNeasy Mini kit (Qiagen # 74124) as per manufacturer specifications. cDNA was generated by oligo-dT or random hexamer priming with either omniscript enzyme (Qiagen # 205111 ), primescript enzyme (Clonetech # 2680A) or revert aid enzyme (Thermo-scientific EP0441) according to manufacturers’ specifications. For RACE PCR and extracts from organisms with high GC content, cDNA was generated by incubating the enzyme at gradually increasing temperature from 42 to 65 °C. First-strand cDNA samples were then incubated with RNaseH (New England Biolabs # M0297S) for RNA degradation prior to use in downstream PCR reactions. RACE PCR: polyA-cDNA was generated by incubating cDNA with terminal deoxynucleotidyl transferase (NEB # M0315S) in presence of dATP (200 μM) for 20 min at 37 °C, and incubation at 70 °C for 10 min.

PCR primers: for 5′RACE PCR, oligo-dT anchored primer was used for initial PCR cycles, GGACTCGAGTCGACATCGATTTTTTTTTTTTTTTTTGG, when nested PCR was required, second amplification required oligo GGACTCGAGTCGACATCGA. This oligonucleotide was also used for 3′RACE after 10 cycles with the primer: GGACTCGAGTCGACATCGATTTTTTTTTTTTTTTTTVN.

Degenerated oligonucleotides to amplify *erk* from dogfish were : >PL-09-10 CCATCAAAAAGATCAGCCCT; >PL-09-11 GGGTCATAGTACTGCTCCAGGTA; for skate > PL-11-23 CTTCTGCAGAGGACCTGAACTG; >PL-11-24 GGACCAGCTCAATCATATCCTTG;>PL-11-25 GCTGGCAGTATGTCTGGTGTTC > PL-11-26 CTCATGTTTGAAGCGCAGCAG; for Bichir *erk*s a combination of oligonucleotides was used for nested PCR and extension of clones > PL-11-19 gtggccatcaagaagatcagccc > PL-11-20 Gagcaccagacwtactgccagcg; >PL-11-42 GACCTGAAGCCGTCCAACC; >PL-11-43 CATCCACTCAGCAAATGTTCTCC;>PL-11-44 CTTTGGATCTGCTGGATCGAATG; >PL-11-49 GATCTGAAACCCTCTAACTTGCTG; >PL-11-50 CAAATCCTGCGAGGCTTAAAGTA. Combinations of primers were used to clone *erk* isoforms from cDNAs, primers for *Crocodylus niloticus erk1* were: >PL-12-07 GCGGCCATCAAGAAGATCAG; >PL-12-08 ATCGGCATCAACGACATCCT; >PL-12-09 GTACATCCACTCGGCCAACGT; >PL-12-10R GCCTTCATGTTGATGATGCAGTT; >PL-12-11R GCTTGTTGGGGTTAAAGGTCA; >PL-12-12R GGTCGTAGTACTGCTCCAGGTAGG; primers for cloning *Trachemys scripta elegans erk1* were: >PL-10-16 GCTGTATCCTGGCGGAGATG; >PL-10-17 ATCATGCTCAACTCCAAGGGCTA; >PL-11-07 GCGGCCTCAAGTACATCCACTCG ; >PL-11-08 GCGATCTCAAGATCTGTGACTTTGGTC ; >PL-11-15 GCGCTACACGGACCTGCAGTACATC ;>PL-11-16 TACACGGACCTGCAGTACAT; >PL-11-17R GAGCTGGTCCAGGTAGTGCTTGCC ; >PL-11-18R CAAGCACTACCTGGACCAG; primers for cloning *Anolis carolinensis erk1* were: >PL-12-16 GTAAAGCAGCAGCAGCACTAAGGA; >PL-12-17 GATTCTCCAGCGGATCGGC; >PL-09-04 TCATCGGCAT CAATGACATT; >PL-09-05 TAAAGACCCAGCAACTCAGCAA; >PL-09-06R GTGTGGTCATGGTCTGGATC; >PL-09-07R CCAGGAAAGATGGGTCGGTT; >PL-10-20 TCCGGGTAAAGCAGCAGCAGC; primers for cloning *Crocodylus niloticus erk2* were: >PL-10-27 CCAACTATTGAGCAAATGAAAGATG; >PL-10-28 CAGCACCTCAGCAACGACCAC; >PL-11-67 CGTGCAAGATCTCATGGAGACAG; >PL-12-13 CTACAGGCTCATCACTTGGATCGTA; >PL-12-14R GGCTGGAATCTAGCAGTTTCCTC; >PL-11-68R CATCTTTCATTTGCTCAATAGTTGG; >PL-11-69R GTGGTCGTTGCTGAGGTGCTG; >PL-11-70R CTGTCTCCATGAGATCTTGCACG; >PL-11-71 GTGGCCTATCTCAGCTTATCCAC; primers for cloning *Anolis carolinensis erk2* were: >PL-12-15 CCTCTTCCTCCTCGCTGTTTC; >PL-09-24 TATTTCCTTTATCAAATCCTGAG; >PL-09-25R CAGCTGATCGAGATAGTGTTTC; >PL-10-21 GAGTTGTGTCTTCCTTCGCCTTCC; >PL-10-22R CCTCTCAGGATYTGATAAAG; >PL-11-11 GCGAGAAATCAAAATCCTCT; >PL-11-12R CGGCACTTTGTTTTTGTAC; pri-mers for cloning *Trachemys scripta elegans erk2* were: >PL-10-23 CGGGGGTCCGGAGATGGT; >PL-10-24 TACGGCATGGTCTGTTCTGCCTA; >PL-11-09 CCACACGTTGGTACAGAGCACCTG; >PL-11-10R AAGTATGGTCAGCAGTGGGTGCAC; >PL-11-06 GAAGTGGAGCAAGCTTTGGC. Fragments subcloned for normalization of absolute RT-qPCR were obtained with the following primers: *Anolis carolinensis erk1* > PL-12-17 GATTCTCCAGCGGATCGGC and > PL-09-07 CCAGGAAAGATGGGTCGGTT *Anolis carolinensis erk2* > PL12-15 CCTCTTCCTCCTCGCTGTTTC and > PL-12-13 CTACAGGCTCATCACTTGGATCGTA; *Trachemys scripta elegans erk1* > PL-11-16 tacacggacctgcagtacat and > PL-09-09 GAGCTGGTCCAGGTAGTGCTTG *Trachemys scripta elegans erk2*: >PL-10-24 TACGGCATGGTCTGTTCTGCCTA and > PL-11-04 GCTTACTAATGCATATGGATGC*; Crocodylus niloticus erk1*: >PL-12-07 GCGGCCATCAAGAAGATCAG and > PL-12-12 GGTCGTAGTACTGCTCCAGGTAGG*; Crocodylus niloticus erk2*: >PL-11-67 CGTGCAAGATCTCATGGAGACAG and > PL-12-13 CTACAGGCTCATCACTTGGATCGTA.

### Quantitative RT-PCR

RNAs were processed as described in the previous section, for quantification of Fig. 4, RNAse-free DNAse was added in solution prior to extracting RNA from columns (Qiagen #79254) and cDNA was generated with nonamers or hexamers priming by Qiagen Omniscript kit as recommanded by supplier; for quantifications of Additional file 4, cDNA was generated with Qiagen Quantiscript kit with nonamers (genomic DNA diminished by g-removal buffer). Quantitative PCR was performed on Applied Biosystems 7300 and Step One plus Real Time PCR System. For sybr green detection, mouse erk1 was amplified with primers > ERK1-03mForward CCTGCTGGACCGGATGTTA and > ERK1-03mReverse TGAGCCAGCGCTTCCTCTAC, mouse erk2 was amplified with primers > ERK2-02mForward GGAGCAGTATTATGACCCAAGTGA > ERK2-02mReverse TCGTCCACTCCATGTCAAACT, anolis erk1 was amplified with primers > anolisERK1-5forward CAAGATTCGAGCTGCCATCA and > anolisERK1-5reverse GCAGTGTGCGTTGGCAATAA, anolis erk2 was amplified with primers > anolisERK2-Cforward AAACAAAGTGCCGTGGAACAG and > anolisERK2-C reverse GGATGGGCCAAAGCTTCTTC. For TaqMan quantification primer mix were from AppliedBiosystems, Mn01973540-g1 for ERK1 and Mn00442479-m1 for ERK2. Quantity of molecules was determined by normalisation with linearized plasmid. For mouse erk1 and erk2, full length cDNAs cloned were used [10]; for A. carolinenis, T. scripta elegans and C. niloticus, the plasmids with partial cDNA were cloned into invitrogen PCR2topo vector (sequences of the primers presented in the section DNA sequences). Dilutions of linearized plasmids ranged from 30 to 300 000 molecules per reaction. Each reaction was performed with cDNAs generated from 25 ng of RNA.

### Phylogeny

ProtTest [39] was used to evaluate the best model to use with the MaximumLikelihood method and the protein sequences for Fig. 2b. The tree obtained with the best model showed little difference with the tree resulting from the consensus of all model. In addition sequences were analyzed using SeaView [40]. Sequences were aligned as amino-acid sequences and then back converted to nucleotide sequences (as implemented in SeaView) and alignments were manually checked. Trees were then built using the BioNJ (Kimura correction), Parsimony and Maximum likelyhood methods as implemented in SeaView.

For computations of Shannon entropy, an ad-hoc Python program was written. For nucleotide sequences, indels were treated as a 5^th^ character. Since *erk1* and *erk2* differ in length, positions that are all indels in one of the gene have an energy of 0; this can be clearly misleading, but it allowed direct comparison of other positions.

For the identification of Site-Specific Positive or Purifying selections, we used the Selecton program, locally compiled under Linux and with default values [41].

For the calculation of global Ka/Ks ratios of Table 2, the mean nucleotide substitution rate, amino acid substitution rate, nonsynonymous substitution rate (Ka), synonymous substitution rate (Ks), and nonsynonymous to synonymous substitution rate ratio (Ka/Ks) were calculated for ERK1 and ERK2 CDS alignments in MEGA 6 [42]. The shark ERK sequences were excluded in this calculation to ensure our calculation for Erk1 and Erk2 covered exactly the same species sampling. 94 mamalian sequences and 28 vertebrate sequences corresponding to the species’ list of Fig. 2 were studied. The Jukes-Cantor correction model [43] was used when calculating nucleotide substitution rate; the Poisson model [44] was used when calculating amino acid substitution rate; and the Nei-Gojobori model [45] was used when calculating Ka and Ks. The mean Ka/Ks value was calculated by calculating Ka/Ks for each pairwise comparison first and then taking the average.

### 3D structures used to generate still images

3D structures solved from ERK crystals were recovered from Protein Data Bank (PDB): 1ERK (rat ERK2) [46]; 4QTB and 4QTA (respectively human ERK1 and ERK2 bound to inhibitor SCH772984) [47]. Sequences were opened with the freely available 3D software Cn3D (from NCBI), and amino-acids to be highlighted were colored with the annotation tool. (http://www.ncbi.nlm.nih.gov/Structure/CN3D/cn3d.shtml). Still images were generated when 3D structure was rotating in the software Cn3D.

For all structures, threonine and tyrosine phosphorylated upon activation by MEK are colored in white; the amino-acids interacting with substrates forming the DEJL(KIM) motif are colored in blue, light blue for docking groove and darker blue for acidic patch; the amino-acids interacting with substrates via the DEF domain (FXFP) are colored in green.

## Additional files

Additional file 1:
**A. Only one difference (ile/leu) between ERK1 and ERK2 among the 23 amino-acids putatively interacting with substrates.** (i-ii) residues shown to be critical in co-crystal structures for substrate binding in mouse ERK2 are colored in blue. Residue differences in aligned mouse ERK1 and ERK2 sequences are shown or boxed in red. (i) Binding domains on ERKs for binding to the DEJL(KIM) motif of substrates (includes the common docking (CD) domain on ERK) [13]; upper sequences: hydrophobic groove of ERKs, lower sequences: acidic patch of ERKs (total of 16 amino-acids that interact directly with substrates). (ii) Domains on ERKs for binding to the DEF (FXFP) motifs of substrates [14], (total of 7 amino-acids that interact with substrates).** B: Mouse and Anolis ERK sequences are highly similar.** ERK1 protein sequences (top) and ERK2 protein sequences (bottom) from *M. musculus* and *A. carolinensis* are aligned side by side with the software multalin (http://multalin.toulouse.inra.fr/multalin/). Amino-acids in red are identical between mouse and anolis sequences, whereas amino-acids in black differ between mouse and anolis sequences. The percentage identity between sequences is calculated with the sequences after the arrow indicated in the figure (the N-terminal end of the molecules which is extremely variable in all vertebrates is not taken into account). **C: 3D structures of human ERK1 and human ERK2 are highly similar, especially at the “face of the kinase”.** Human ERK1 and human ERK2 were aligned by multalin program, and amino-acids that diverge between ERK1 and ERK2 are highlighted in yellow on 3D human ERK1 structure (PDB #4QTB). At ERK1 positions in the face of the kinase, the ERK2 equivalent amino-acids are indicated after the yellow arrows. Docking sites, activation phosphorylation sites and the dashed white line circling the “face of the kinase” are colored as presented for Fig. 5. i) front-side of the kinase where ATP-transfer occurs in the catalytic cleft between the two lobes ii) left-side of the kinase iii) back-side of the kinase iv) right-side of the kinase. **D: 3D structure of anole lizard ERK2 is highly similar to rat ERK2.** The amino-acid divergent between anole lizard ERK2 and human ERK2 are highlighted in yellow on 3D rat ERK2 structure (PDB #1ERK). Docking sites, activation phosphorylation sites and the dashed white line circling the “face of the kinase” are colored as presented for Fig. 5. E: Structures of anole lizard ERK1 is highly similar to human ERK1. The amino-acid divergent between anole lizard ERK1 and human ERK1 are highlighted in yellow on 3D human ERK1 structure (PDB #4QTB). Docking sites, activation phosphorylation sites and the dashed white line circling the “face of the kinase” are colored as presented for Fig. 5. The names of amino-acids of the “face of the kinase” that diverge are named in yellow, with the corresponding amino-acid of anole lizard named after the yellow arrows (differences occur only for conserved substitutions: serine/threonine and glutamic acid/aspactic acid). (PDF 2230 kb) Additional file 2:
**ERK1 and ERK2 protein expression in brain areas. (A-D) Brain areas were dissected as presented in Materials and Methods.** Upper immunoblot: incubated with anti-ERK antibody, lower immunoblot: incubated with anti-phospho-ERK antibody. Positions of ERK1, ERK2, phospho-ERK1 (pERK1) and phospho-ERK2 (pERK2) are indicated on the sides. Brain parts are CE: cerebellum, CO: cortex, CR: cerebellum white matter center, PG: pituitary gland, ME: medulla, MS: mesencephalon, Mix: bottom of the brain in cranial box, OP: optic lobe, OL: olfactory bulb, PI: pineal gland, SC: spinal cord, TE: telencephalon. Control extracts from cultured cells, CCL39 from Chinese hamster (*Cricetulus griseus*) or NIH3T3 from mouse (*Mus musculus*); or from cattle medulla (*Bos Taurus*) are loaded on immuno-blots sides. (A) teleosts (European seabass, *Dicentrarchus labrax*, and African jewelfish, *Hemichromis bimaculatus*), and Bichir (*Polypterus senegalus*)*.* (B) Amphibians: African clawed frogs (*Xenopus laevi*) and Axolotl (*Ambiostoma mexicanum*). (C) Birds: chicken (*Gallus gallus*) and Greater rhea (*Rea americana*). (D) Mammals: European rabbit (*Oryctolagus cuniculus*), cattle (*Bos taurus*) and the marsupial, Gray short-tailed opossum (*Monodelphis Domestica*). (PDF 2231 kb) Additional file 3:
**A. The epitope of the antibody targeting active ERK is conserved from sea anemone to human.** Alignment of ERK1/2 residues which surround the sites phosphorylated by MEK kinase. The sequence HTGFLTRYVAT corresponds to the epitope recognized by the monoclonal anti-phosho ERK antibody from Sigma (#M8159). Phosphorylation sites, threonine, and tyrosine are indicated by arrows and are highlighted in yellow. ERK1 list in red, ERK2 list in blue, and single ERK list in black. Species names, sequences, accession numbers and common names are presented in that order. Note: yeast sequences of the two ERK orthologous genes, Fus3 and Kss1 are presented to illustrate that the phospho-ERK epitope is not fully conserved in yeast, unlike in sea anemone for example. **B**: **Three distinct antibodies recognize phosphorylated ERK in extracts from mouse, lizard and hagfish.** 10 % acrylamide gels were loaded with protein samples from mouse fibroblasts NIH3T3 or lizard embryo fibroblasts (*A. sageii* and *A. carolinensis*) or hagfish brain extracts as indicated in the figure (top). Mouse fibroblasts were pre-treated for one hour with 20 μM UO126 prior cell lysis to block activation of the ERK signaling cascade (lane1), or were stimulated with 10 % serum in presence of 1 mM sodium orthovanadate (NaVO^4--^) for one hour to block phosphatases and activate maximally the ERK signaling cascade (lanes 2). Lizard fibroblasts, from A. carolinensis (lane 3) or A. sagreii (lane 4) were stimulated as mouse fibroblasts of lane 2. One gel was stained with coomassie blue (i), other gels were transferred onto nitrocellulose membrane as described in materials and methods and immunoblots performed by incubating in the following antibodies ii) Anti phospho-ERKs sigma #M7802 iii) Anti phospho-ERKs Cell signaling #9101 iv) Anti phospho-ERKs Abcam #32538 v) Anti total-ERKs from Cell-Signaling #9102 and vi) Anti total-ERKs homemade E1B [10]. (PDF 2233 kb) Additional file 4:
**erk2 mRNA is more expressed than erk1 mRNA in all mouse tissues tested.** RNA was purified and cDNA was generated from nonamers primers with quantitec kit from qiagen as described in materials and methods. Similar results were obtained by RT-PCR with primers sybr green or taqman probe detection (materials and methods). (PDF 2229 kb) Additional file 5:
**Phylograms of ERK nucleic or protein sequences in vertebrates and sequence divergences. Phylogenetic analysis of erk nucleotides sequences (A,C) and derived ERK amino acid sequences (B,D) from organisms at key evolutionary nodes in the vertebrate linage by neighbor joining analysis (A,B) and maximum parsimony (C,D).** erk1/ERK1 (mapk3/MAPK3, red branches) erk2/ERK2 sequences (mapk1/MAPK1, blue branches). Tetrapods or Chondrichthyes are indicated by arrows or brackets. Accession numbers and common names are listed in Figure 7. (E,F) Shannon entropy for erk sequences. A multiple sequence alignment was conducted on all available full-length ERK1/*erk1* and ERK2*/erk2* pairs of mammalian sequences (12 *pairs*) and a .fasta file was constructed for each gene using the conserved domains determined by the alignment. (E) Abscissa: distance from start ATG codon (F) Abscissa: distance from starting mRNA nucleotide. Ordinate: calculated Shannon entropy values for each position, amino-acid (E) and nucleotide (F). Arrows on the side indicate that the lower entropy indicate greater divergences among sequences at that residue position. In the 12 mammals analyzed, ERK1 proteins diverge at 42 positions whereas ERK2 proteins diverge at only 11 positions. Furthermore, without taking into account the extreme N-terminal poly-alanine stretch, the difference is wider: 35 positions diverge among ERK1s versus 4 positions among ERK2s. (PDF 2242 kb) Additional file 6:
**ERK1 and ERK2 protein expression in reptiles. (A to C) presentations as in Additional file 3.** (A) Cerebellum samples from lizard (A. Carolinensis), crocodile (C. niloticus) and turtle (T. scripta elegans), control extracts from cattle cortex (Bos Taurus) are loaded on first lane. (B) samples from gecko (T. mauritanica); lizard (A. Carolinensis); snake (T. elegans; mouse NIH3T3 were stimulated for 10 min prior to lysis (lane1) or were stimulated one hour in presence of MEK inhibitor PD184352 (lane2) to block ERK phosphorylation by MEK Kinase. (C) Triton lysis of different lizard extracts (A. Carolinensis) was performed as described in materials and methods, 10 % acrylamide gels were loaded with 15 μg protein. Extract from mouse NIH3T3 fibroblasts (stimulated for one hour with sodium-orthovanadate and 10 % serum) was loaded in lane 1 as control. Upper panel: coomassie staining illustrates overall uniform protein loading of samples despite great differences in expression of major abundant proteins. Median panel: anti total-ERK antibody reveals ERK1 and ERK2 proteins in mouse and only ERK1 protein in all anolis tissues. Lower panel: anti-phospho ERK antibody confirms that only one ERK is expressed in all anolis tissues tested. Note that despite triton lysis and centrifugation of insoluble material, major proteins produce some non-specific binding. (D) Full species names of reptiles whose brains were dissected in the laboratory for this study. (PDF 2230 kb) Additional file 7:
**Animal names and accession numbers of erk cDNA sequences analyzed in the present study.** **(A)** List of vertebrate sequences used in Figure 2A, Figure 2B and Additional Figure 5A-D. **﻿﻿﻿﻿(B)** list of 37 mammalian pairs of ERKs whose sequences were used for Fig. 5. **(C)** list of 12 mammalian pairs of full-length ERKs sequences, used for Additional Figure 5E-F. **(D)** Correspondence between taxonomic and common names of the animals studied. list of 37 mammalian pairs of ERKs whose sequences were used for Figure S4C and D. list of 12 mammalian pairs of full-length ERKs sequences, used for Figure S7. taxonomic names of animals studied in this work. (PDF 2233 kb)
