# Supplementary material for: ERK1 and ERK2 present functional redundancy in tetrapods despite higher evolution rate of ERK1
Source: BMC Evol Biol. 2015 Sep 3;15:179. doi: 10.1186/s12862-015-0450-x (PMC4559367; doi:10.1186/s12862-015-0450-x)

**Additional file 1 A**

**i**

hydrophobic groove of ERKs

**ERK1** $^{127}$ETDLYKLLKSQQLSNDHICYFLY$^{149}$ $^{175}$INTTCD$^{180}$
**ERK2** $^{107}$ETDLYKLLKTQHLSNDHICYFLY$^{129}$ $^{155}$LNTTCD$^{160}$

acicid patch of ERKs

**ERK1** $^{97}$RHENI$^{101}$ $^{141}$NDHICYFLYQILR$^{153}$ $^{177}$TTCD$^{180}$ $^{335}$YDPTDE$^{340}$
**ERK2** $^{77}$RHENI$^{81}$ $^{121}$NDHICYFLYQILR$^{133}$ $^{157}$TTCD$^{160}$ $^{315}$YDPSDE$^{320}$

**ii**

**ERK1** $^{213}$YRAPEIMLNSK$^{221}$ $^{248}$GKHYLDQLNHI$^{258}$ $^{278}$ARNYLQS$^{284}$
**ERK2** $^{193}$YRAPEIMLNSK$^{201}$ $^{228}$GKHYLDQLNHI$^{238}$ $^{258}$ARNYLLS$^{264}$

**Additional file 1 B**

Alignment of ERK1 sequences (MAPK3) from A.carolinensis and M. musculus
96.9 % identity after variable N-term (33 differences out of 351 aa)

```
                1                                                                                    80
A.caro ERK1  MSAATAAAPG GAGAVPVPGA PRLGV----- ETVKGQVFDV GPRYTDLQYI GEGAYGMVCS SYDHVNKIRA AIKKISPFEH
M.musc ERK1   MAAAAAAPG GGGGEPRGTA GVVPVVPGEV EVVKGQPFDV GPRYTQLQYI GEGAYGMVSS AYDHVRKTRV AIKKISPFEH

             81                                                                                 160
A.caro ERK1  QTYCQRTLRE IKILLRFKHE NVIGINDILR APTIDQMRDV YIVQDLMETD LYKLLKTQQL SNDHICYFLY QILRGLKYIH
M.musc ERK1  QTYCQRTLRE IQILLRFRHE NVIGIRDILR APTLEAMRDV YIVQDLMETD LYKLLKSQQL SNDHICYFLY QILRGLKYIH

             161                                                                                240
A.caro ERK1  SANVLHRDLK PSNLLINTTC DLKICDFGLA RIADPDHDHT GFLTEYVATR WYRAPEIMLN SKGYTKSIDI WSVGCILAEM
M.musc ERK1  SANVLHRDLK PSNLLINTTC DLKICDFGLA RIADPEHDHT GFLTEYVATR WYRAPEIMLN SKGYTKSIDI WSVGCILAEM

             241                                                                                320
A.caro ERK1  LSNRPIFPGK HYLDQLNHIL GILGSPSQDD LNCIINMKAR NYLQSLPQKP KVPWNKLFPK ADPKALDLLD KMLTFNPNKR
M.musc ERK1  LSNRPIFPGK HYLDQLNHIL GILGSPSQED LNCIINMKAR NYLQSLPSKT KVAWAKLFPK SDSKALDLLD RMLTFNPNKR

             321                                                      381
A.caro ERK1  ITVEESLAHP YLEQYYDPSD EPVAEEPFTF DMELDDLPKE KLKELIFEET ARFQPGYQGP
M.musc ERK1  ITVEEALAHP YLEQYYDPTD EPVAEEPFTF DMELDDLPKE RLKELIFQET ARFQPGAPEG P
```

Alignement of ERK2 sequences (MAPK1) from A.carolinensis and M. musculus
96.9 % identity after variable N-term (11 differences out of 351 aa)

```
             1                                                                                   80
A.caro ERK2  MAAVSGAAGG SGANPAGGPE MVRGQAFDVG PRYTNLSYIG EGAYGMVCSA YDNVNKVRVA IKKISPFEHQ TYCQRTLREI
M.musc ERK2             MAAAAAAGPE MVRGQVFDVG PRYTNLSYIG EGAYGMVCSA YDNLNKVRVA IKKISPFEHQ TYCQRTLREI

             81                                                                                 160
A.caro ERK2  KILLRFKHEN IIGINDIIRA PTTDQMKDVY IVQDLMETDL YKLLKTQHLS NDHICYFLYQ ILRGLKYIHS ANVLHRDLKP
M.musc ERK2  KILLRFRHEN IIGINDIIRA PTIEQMKDVY IVQDLMETDL YKLLKTQHLS NDHICYFLYQ ILRGLKYIHS ANVLHRDLKP

             161                                                                                240
A.caro ERK2  SNLLLNTTCD LKVCDFGLAR VADPDHDHTG FLTEYVATRW YRAPEIMLNS KGYTKSIDIW SVGCILAEML SNRPIFPGKH
M.musc ERK2  SNLLLNTTCD LKICDFGLAR VADPDHDHTG FLTEYVATRW YRAPEIMLNS KGYTKSIDIW SVGCILAEML SNRPIFPGKH

             241                                                                                320
A.caro ERK2  YLDQLNHILG ILGSPSQEDL NCIINLKARN YLLSLPYKNK VPWNRLFPNA DAKALDLLDK MLTFNPHKRI EVEEALAHPY
M.musc ERK2  YLDQLNHILG ILGSPSQEDL NCIINLKARN YLLSLPHKNK VPWNRLFPNA DSKALDLLDK MLTFNPHKRI EVEQALAHPY

             321                                           368
A.caro ERK2  LEQYYDPSDE PVAEAPFKFD MELDDLPKEK LKELIFEETA RFQPGYRC
M.musc ERK2  LEQYYDPSDE PIAEAPFKFD MELDDLPKEK LKELIFEETA RFQPGYRS
```

Additional file 1 C

**Differences between human-ERK1 and human-ERK2**

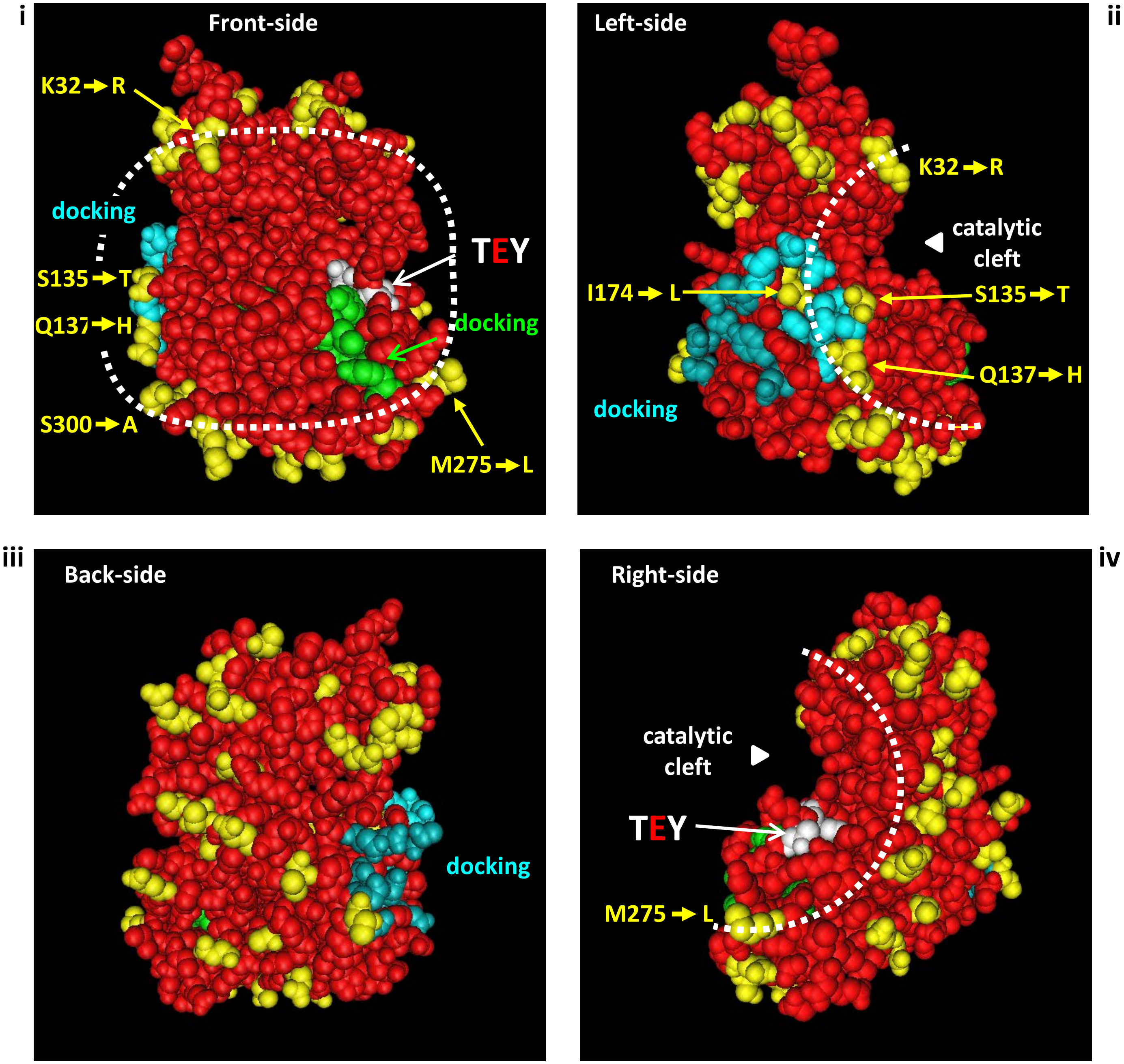

Additional file 1 D

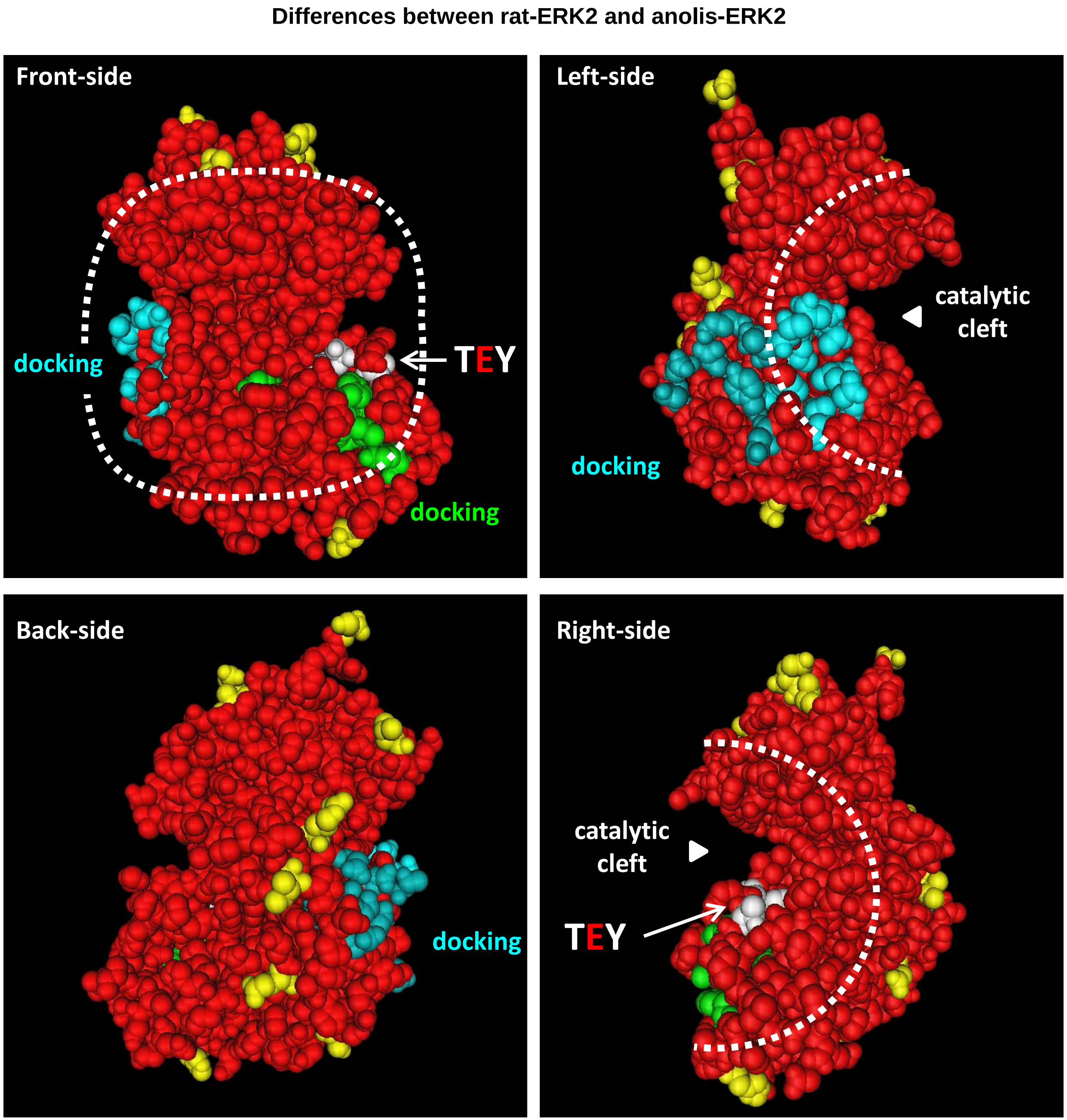

Additional file 1 E

Differences between human-ERK1 and anolis-ERK1

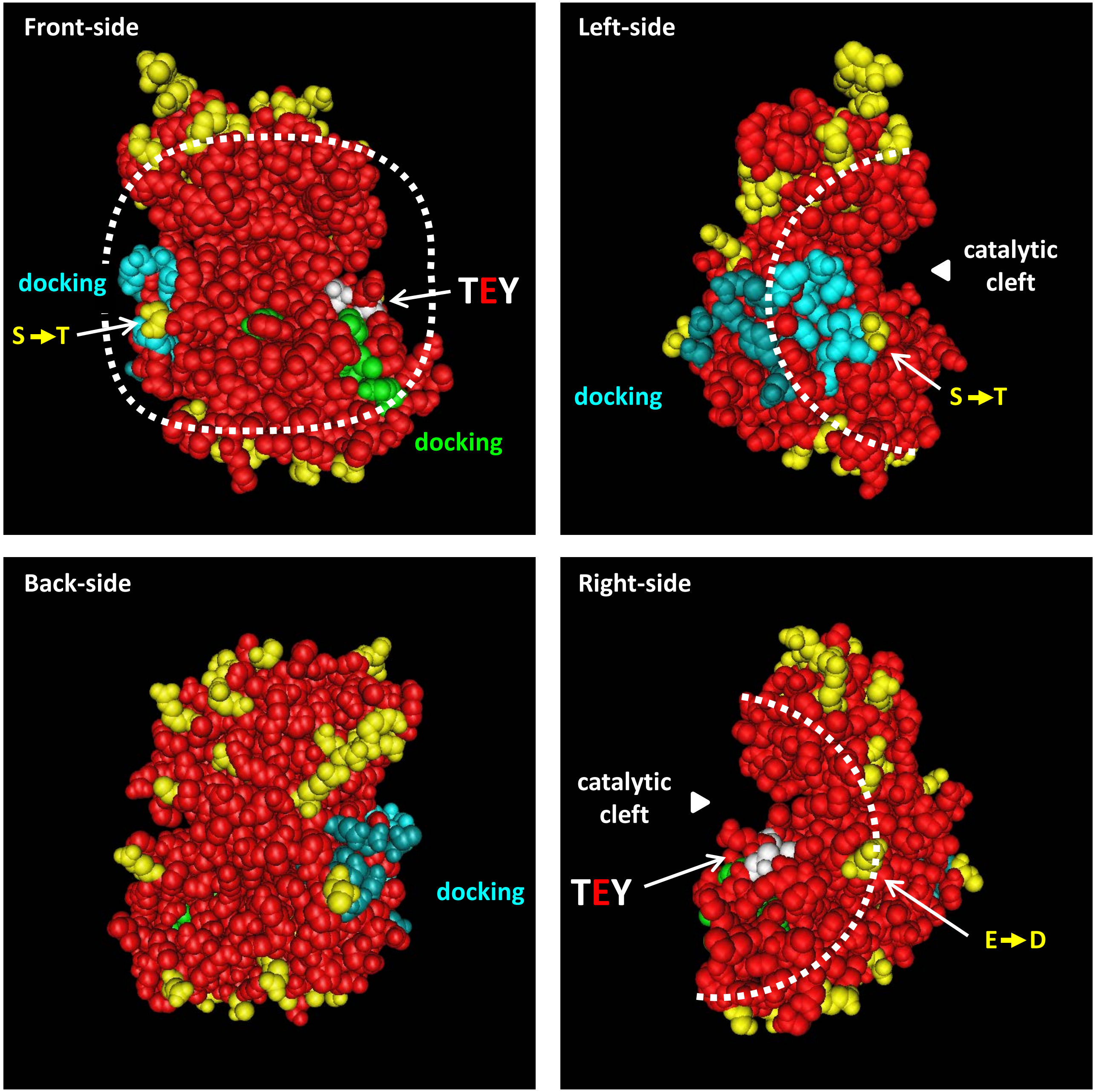

Supplement: Additional file 1: — A. Only one difference (ile/leu) between ERK1 and ERK2 among the 23 amino-acids putatively interacting with substrates. (i-ii) residues shown to be critical in co-crystal structures for substrate binding in mouse ERK2 are colored in blue. Residue differences in aligned mouse ERK1 and ERK2 sequences are shown or boxed in red. (i) Binding domains on ERKs for binding to the DEJL(KIM) motif of substrates (includes the common docking (CD) domain on ERK) [13]; upper sequences: hydrophobic groove of ERKs, lower sequences: acidic patch of ERKs (total of 16 amino-acids that interact directly with substrates). (ii) Domains on ERKs for binding to the DEF (FXFP) motifs of substrates [14], (total of 7 amino-acids that interact with substrates). B: Mouse and Anolis ERK sequences are highly similar. ERK1 protein sequences (top) and ERK2 protein sequences (bottom) from M. musculus and A. carolinensis are aligned side by side with the software multalin (http://multalin.toulouse.inra.fr/multalin/). Amino-acids in red are identical between mouse and anolis sequences, whereas amino-acids in black differ between mouse and anolis sequences. The percentage identity between sequences is calculated with the sequences after the arrow indicated in the figure (the N-terminal end of the molecules which is extremely variable in all vertebrates is not taken into account). C: 3D structures of human ERK1 and human ERK2 are highly similar, especially at the “face of the kinase”. Human ERK1 and human ERK2 were aligned by multalin program, and amino-acids that diverge between ERK1 and ERK2 are highlighted in yellow on 3D human ERK1 structure (PDB #4QTB). At ERK1 positions in the face of the kinase, the ERK2 equivalent amino-acids are indicated after the yellow arrows. Docking sites, activation phosphorylation sites and the dashed white line circling the “face of the kinase” are colored as presented for Fig. 5. i) front-side of the kinase where ATP-transfer occurs in the catalytic cleft between the two [file 12862_2015_450_MOESM1_ESM.pdf]
