# Supplementary figures and images for: ERK1 and ERK2 present functional redundancy in tetrapods despite higher evolution rate of ERK1
Source: BMC Evol Biol. 2015 Sep 3;15:179. doi: 10.1186/s12862-015-0450-x (PMC4559367; doi:10.1186/s12862-015-0450-x)

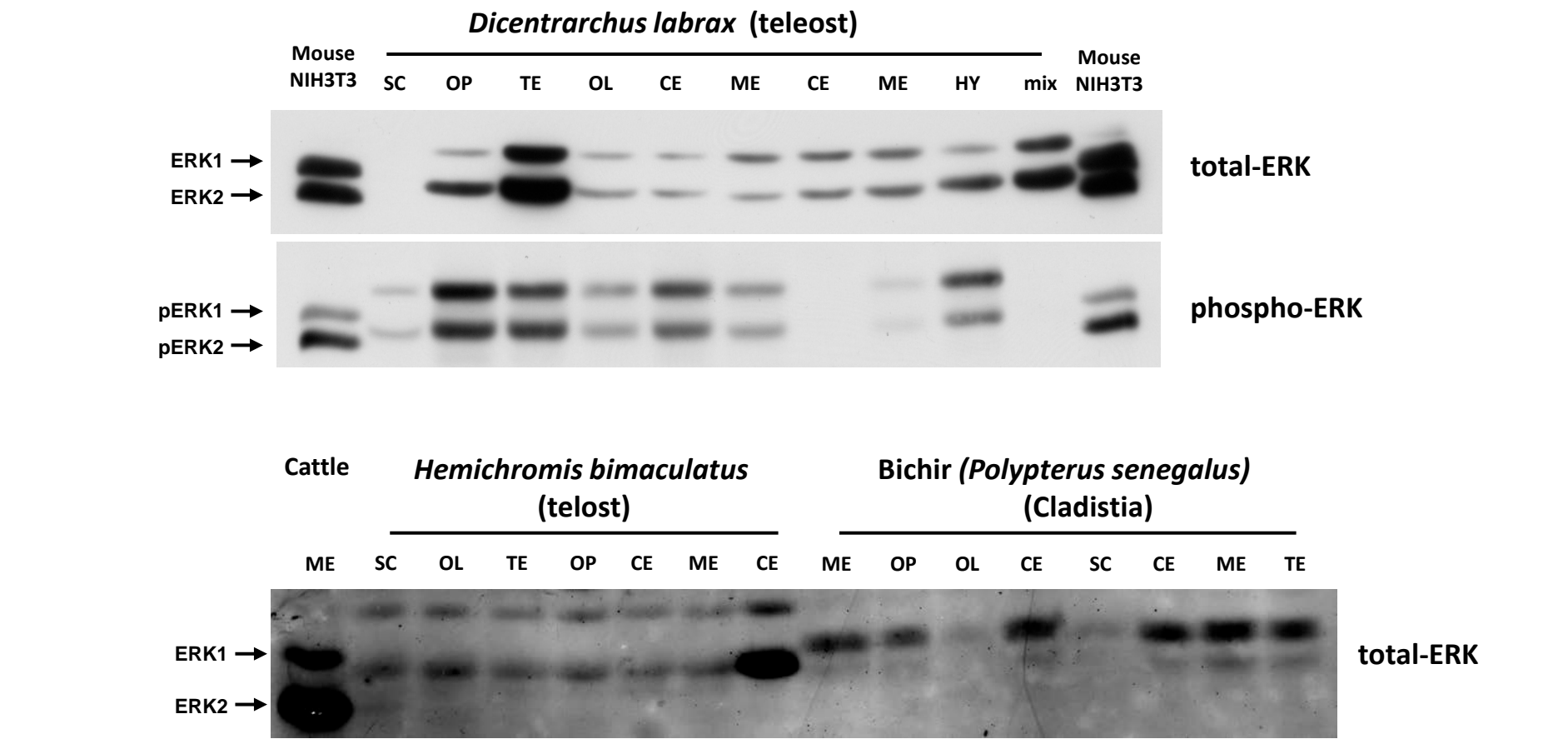

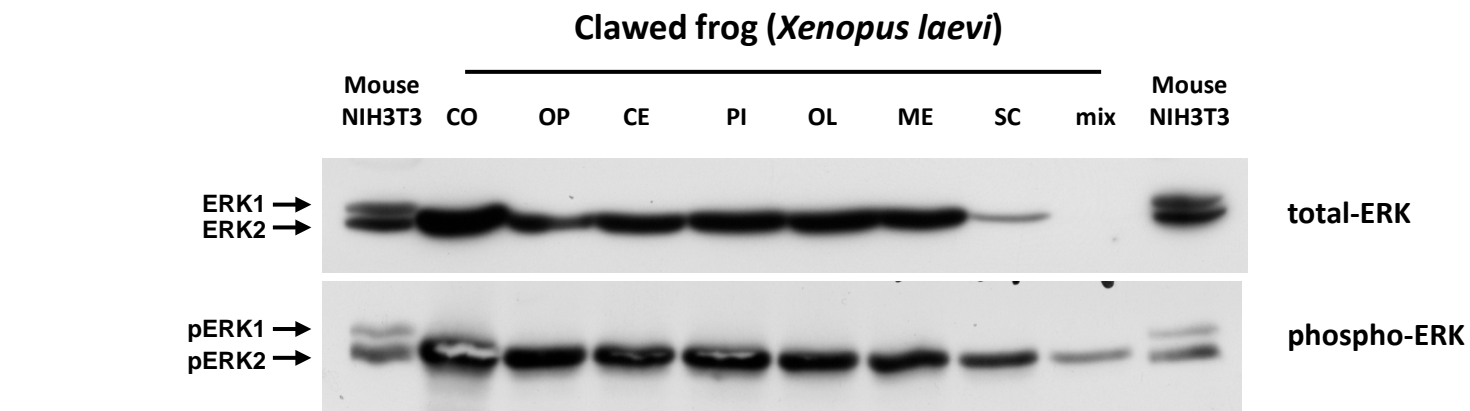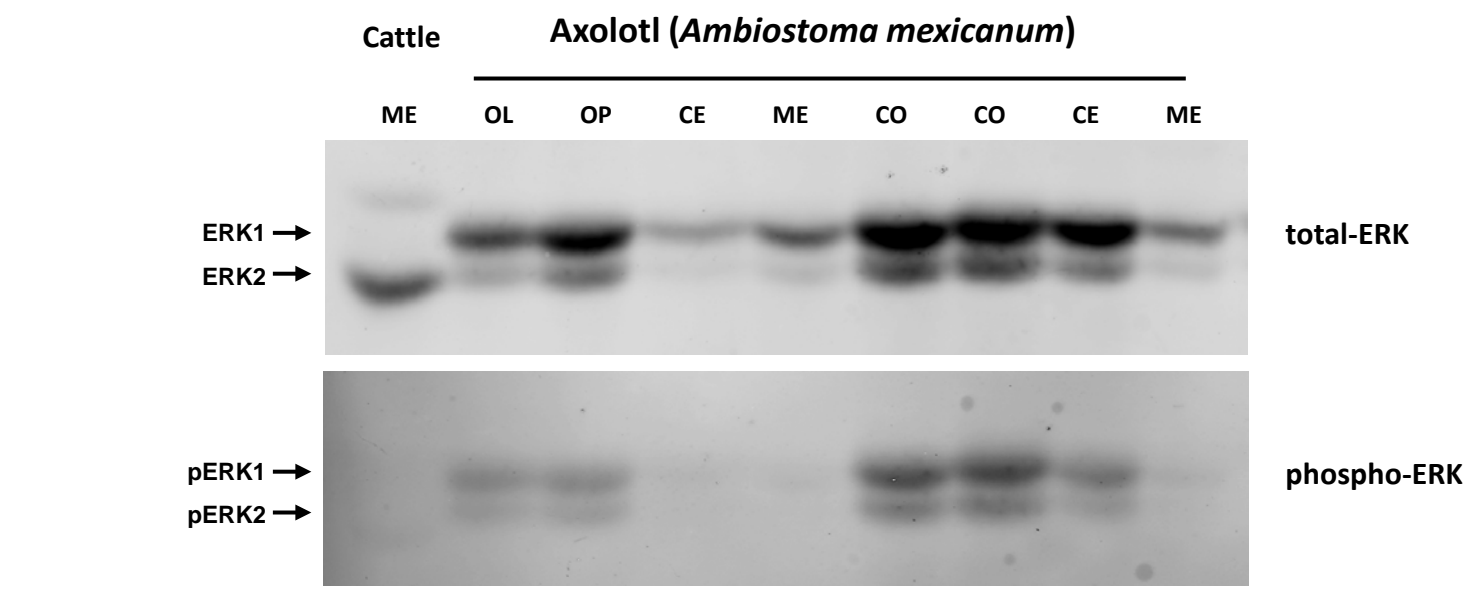

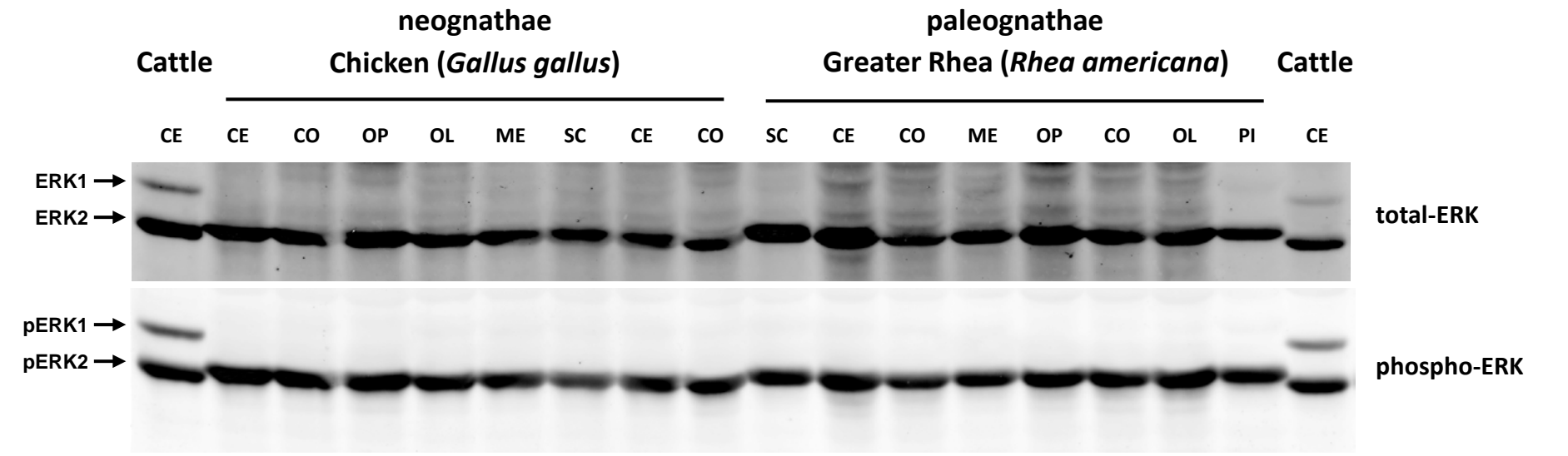

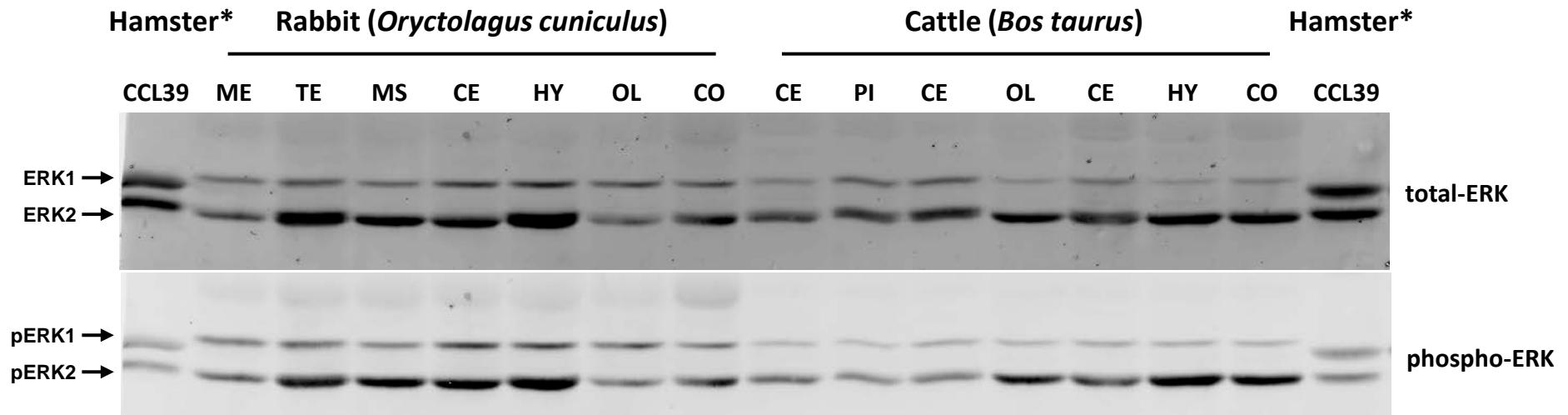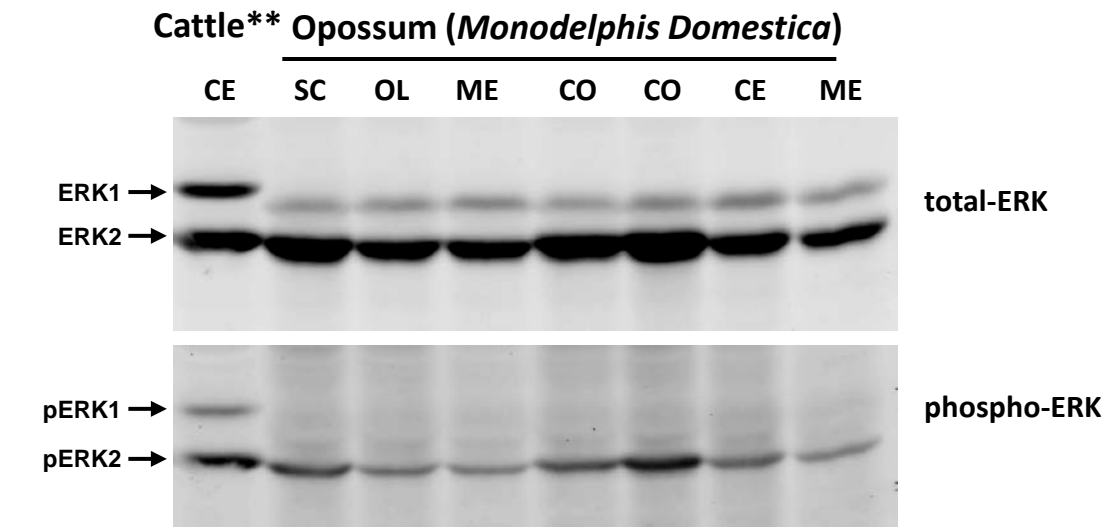

\*Chinese Hamster (*Cricetulus griseus*)  
\*\* Cattle (*Bos taurus*)

Supplement: Additional file 2: — ERK1 and ERK2 protein expression in brain areas. (A-D) Brain areas were dissected as presented in Materials and Methods. Upper immunoblot: incubated with anti-ERK antibody, lower immunoblot: incubated with anti-phospho-ERK antibody. Positions of ERK1, ERK2, phospho-ERK1 (pERK1) and phospho-ERK2 (pERK2) are indicated on the sides. Brain parts are CE: cerebellum, CO: cortex, CR: cerebellum white matter center, PG: pituitary gland, ME: medulla, MS: mesencephalon, Mix: bottom of the brain in cranial box, OP: optic lobe, OL: olfactory bulb, PI: pineal gland, SC: spinal cord, TE: telencephalon. Control extracts from cultured cells, CCL39 from Chinese hamster (Cricetulus griseus) or NIH3T3 from mouse (Mus musculus); or from cattle medulla (Bos Taurus) are loaded on immuno-blots sides. (A) teleosts (European seabass, Dicentrarchus labrax, and African jewelfish, Hemichromis bimaculatus), and Bichir (Polypterus senegalus). (B) Amphibians: African clawed frogs (Xenopus laevi) and Axolotl (Ambiostoma mexicanum). (C) Birds: chicken (Gallus gallus) and Greater rhea (Rea americana). (D) Mammals: European rabbit (Oryctolagus cuniculus), cattle (Bos taurus) and the marsupial, Gray short-tailed opossum (Monodelphis Domestica). (PDF 2231 kb) [file 12862_2015_450_MOESM2_ESM.pdf]

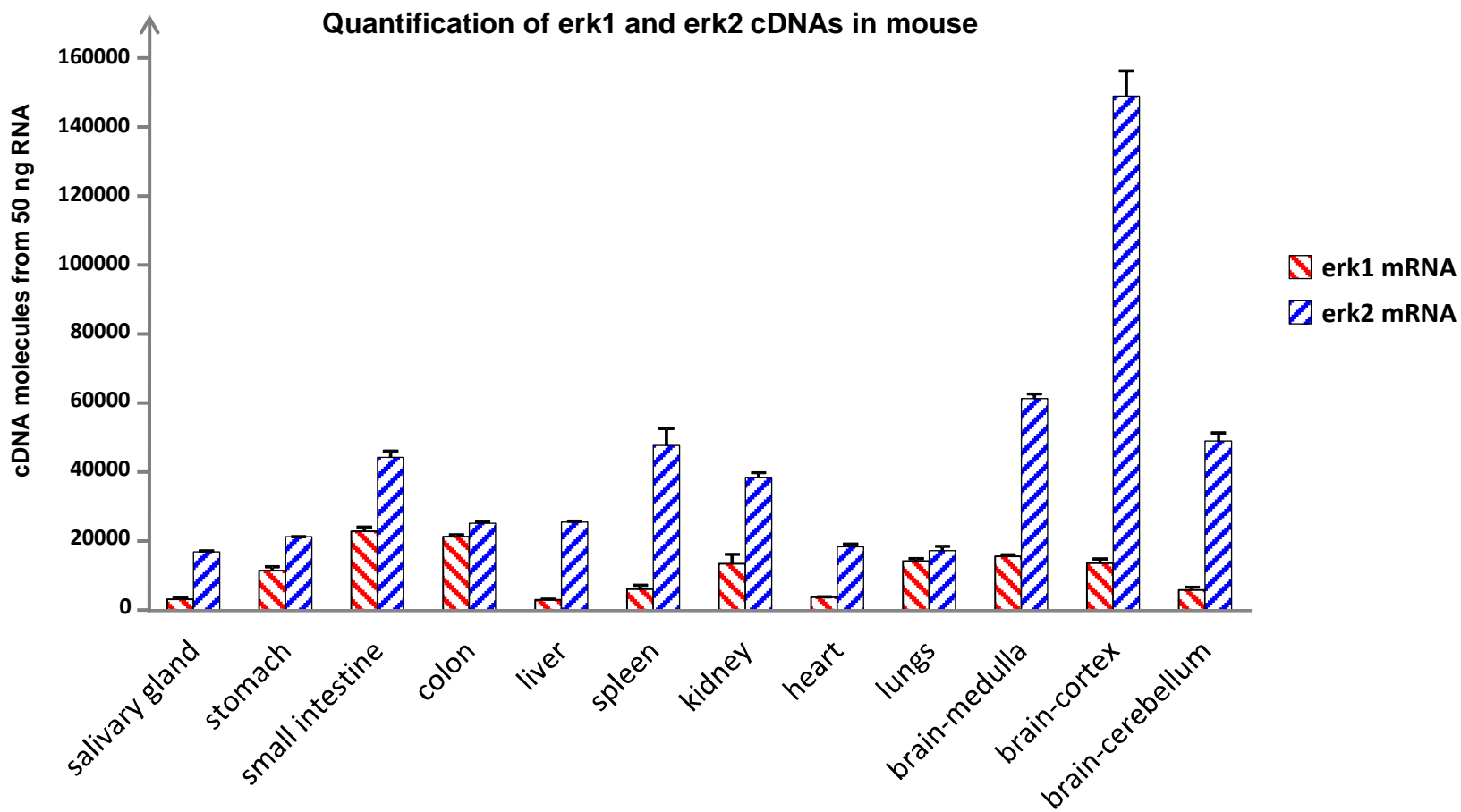

Supplement: Additional file 4: — erk2 mRNA is more expressed than erk1 mRNA in all mouse tissues tested. RNA was purified and cDNA was generated from nonamers primers with quantitec kit from qiagen as described in materials and methods. Similar results were obtained by RT-PCR with primers sybr green or taqman probe detection (materials and methods). (PDF 2229 kb) [file 12862_2015_450_MOESM4_ESM.pdf]
