# Supplementary material for: ERK1 and ERK2 present functional redundancy in tetrapods despite higher evolution rate of ERK1
Source: BMC Evol Biol. 2015 Sep 3;15:179. doi: 10.1186/s12862-015-0450-x (PMC4559367; doi:10.1186/s12862-015-0450-x)

Additional file 3 A

	Species names						MEK phosphorylation site ↓		MEK phosphorylation site ↓				Accession numbers	common names
ERK1	*Homo sapiens*	H	T	G	F	L	T	E	Y	V	A	T	NM_002746.2	Human
ERK1	*Mus musculus*	H	T	G	F	L	T	E	Y	V	A	T	NM_011952.2	Mouse
ERK1	*Bos taurus*	H	T	G	F	L	T	E	Y	V	A	T	XM_005224919.1	Cattle
ERK1	*Dasypus novemcinctus*	H	T	G	F	L	T	E	Y	V	A	T	XM_004461476.1	Armadillo
ERK1	*Monodelphis domestica*	H	T	G	F	L	T	E	Y	V	A	T	XM_001364326.1	Opossum
ERK1	*Anolis carolinensis*	H	T	G	F	L	T	E	Y	V	A	T	XM_003225001.1	Anole lizard
ERK1	*Ambystoma mexicanum*	H	T	G	F	L	T	E	Y	V	A	T	C0317283	Mexican salamander
ERK1	*Takifugu rubripes*	H	T	G	F	L	T	E	Y	V	A	T	XM_003964469.1	Fugu
ERK1	*Danio rerio*	H	T	G	F	L	T	E	Y	V	A	T	NM_201507.1	Zebrafish
ERK1	*Polypterus senegalus*	H	T	G	F	L	T	E	Y	V	A	T	PL-01	Bichir
ERK2	*Homo sapiens*	H	T	G	F	L	T	E	Y	V	A	T	NM_002745.4	Human
ERK2	*Mus musculus*	H	T	G	F	L	T	E	Y	V	A	T	XM_006522147.1	Mouse
ERK2	*Bos taurus*	H	T	G	F	L	T	E	Y	V	A	T	NM_175793.2	Catlle
ERK2	*Dasypus novemcinctus*	H	T	G	F	L	T	E	Y	V	A	T	XM_004466461.1	Armadillo
ERK2	*Monodelphis domestica*	H	T	G	F	L	T	E	Y	V	A	T	XM_001378172.3	Opossum
ERK2	*Gallus gallus*	H	T	G	F	L	T	E	Y	V	A	T	NM_204150.1	Chicken
ERK2	*Anolis carolinensis*	H	T	G	F	L	T	E	Y	V	A	T	XM_003225115.1	Anole lizard
ERK2	*Xenopus tropicalis*	H	T	G	F	L	T	E	Y	V	A	T	NM_001017127.2	Clawed frog
ERK2	*Ambystoma mexicanum*	H	T	G	F	L	T	E	Y	V	A	T	C0314240	Mexican salamander
ERK2	*Takifugu rubripes*	H	T	G	F	L	T	E	Y	V	A	T	XM_003975069.1	Fugu
ERK2	*Danio rerio*	H	T	G	F	L	T	E	Y	V	A	T	NM_182888.2	Zebrafish
ERK2	*Polypterus senegalus*	H	T	G	F	L	T	E	Y	V	A	T	PL-02	Bichir
ERK2	*Callorhinchus milii*	H	T	G	F	L	T	E	Y	V	A	T	SINCAMG00000013172	Elephant shark
ERK	*Petromyzon marinus*	H	T	G	F	L	T	E	Y	V	A	T	ENSPMAG00000009252	Lamprey
ERK	*Eptatretus stoutii*	H	T	G	F	L	T	E	Y	V	A	T	PL-05	Hagfish
ERK	*Ciona savignyi*	H	T	G	F	L	T	E	Y	V	A	T	ENSCSAVT00000014589	Tunicate
ERK	*Branchiostoma lanceolatum*	H	T	G	F	L	T	E	Y	V	A	T	XM_002590654.1	Lancelet
ERK	*Drosophila melanogaster*	H	T	G	F	L	T	E	Y	V	A	T	EYR77323.1	Fly
ERK	*Anopheles gambiae*	H	T	G	F	L	T	E	Y	V	A	T	XM_319983.4	Mosquitoe
ERK	*Caenorhabditis elegans*	H	T	G	F	L	T	E	Y	V	A	T	U27124.1	Worm
ERK	*Nematostella vectensis*	H	T	G	F	L	T	E	Y	V	A	T	XM_001629448.1	Sea anemone

Fus3	*Saccharomyces cerevisiae*	Q	Q	S	G	M	T	E	Y	V	A	T	NM_001178256.1	yeast
Kss1	*Saccharomyces cerevisiae*	L	V	G	F	M	T	E	Y	V	A	T	DQ115391.1	yeast

Additional file 3 B

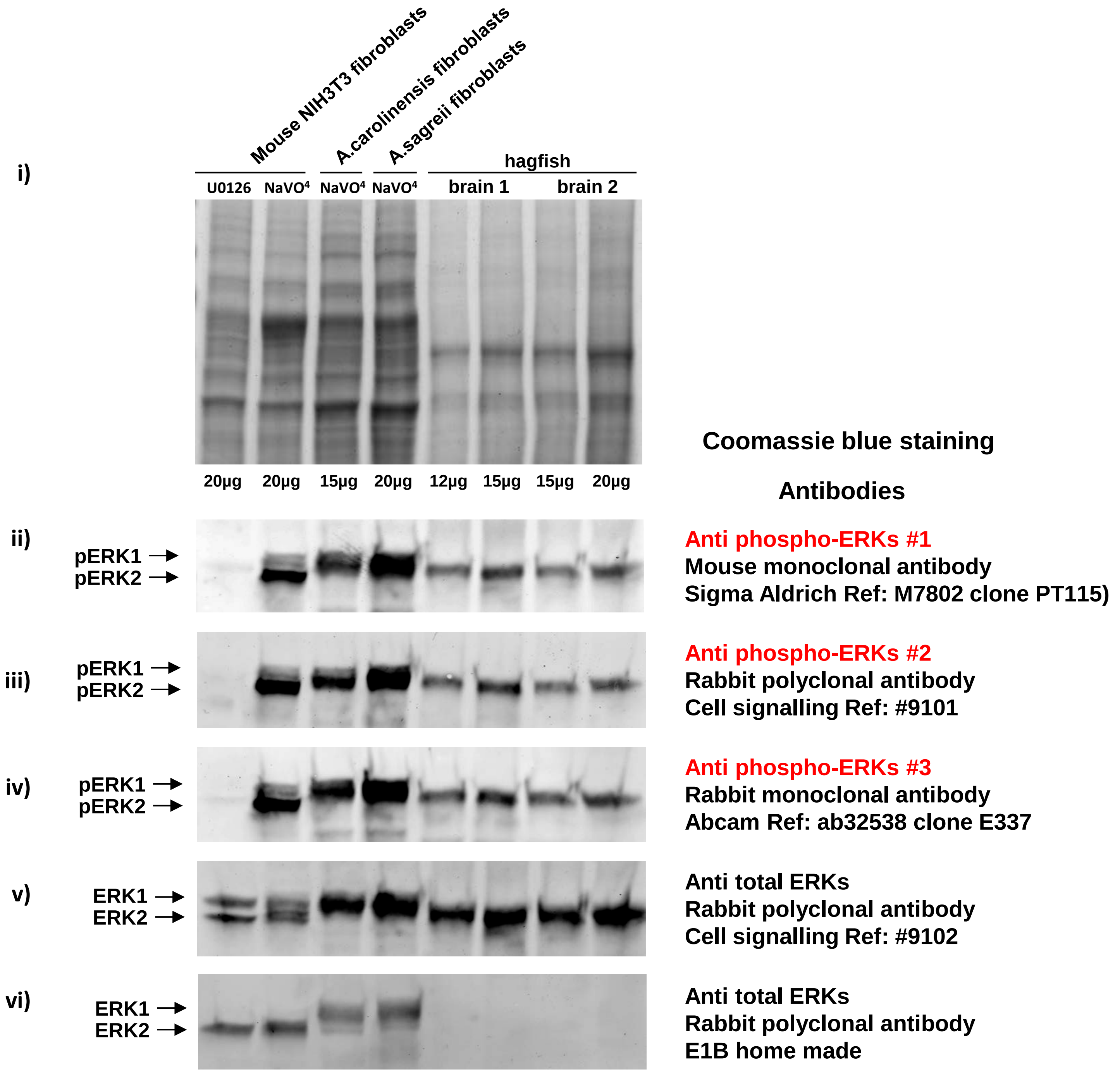

Supplement: Additional file 3: — A. The epitope of the antibody targeting active ERK is conserved from sea anemone to human. Alignment of ERK1/2 residues which surround the sites phosphorylated by MEK kinase. The sequence HTGFLTRYVAT corresponds to the epitope recognized by the monoclonal anti-phosho ERK antibody from Sigma (#M8159). Phosphorylation sites, threonine, and tyrosine are indicated by arrows and are highlighted in yellow. ERK1 list in red, ERK2 list in blue, and single ERK list in black. Species names, sequences, accession numbers and common names are presented in that order. Note: yeast sequences of the two ERK orthologous genes, Fus3 and Kss1 are presented to illustrate that the phospho-ERK epitope is not fully conserved in yeast, unlike in sea anemone for example. B: Three distinct antibodies recognize phosphorylated ERK in extracts from mouse, lizard and hagfish. 10 % acrylamide gels were loaded with protein samples from mouse fibroblasts NIH3T3 or lizard embryo fibroblasts (A. sageii and A. carolinensis) or hagfish brain extracts as indicated in the figure (top). Mouse fibroblasts were pre-treated for one hour with 20 μM UO126 prior cell lysis to block activation of the ERK signaling cascade (lane1), or were stimulated with 10 % serum in presence of 1 mM sodium orthovanadate (NaVO4--) for one hour to block phosphatases and activate maximally the ERK signaling cascade (lanes 2). Lizard fibroblasts, from A. carolinensis (lane 3) or A. sagreii (lane 4) were stimulated as mouse fibroblasts of lane 2. One gel was stained with coomassie blue (i), other gels were transferred onto nitrocellulose membrane as described in materials and methods and immunoblots performed by incubating in the following antibodies ii) Anti phospho-ERKs sigma #M7802 iii) Anti phospho-ERKs Cell signaling #9101 iv) Anti phospho-ERKs Abcam #32538 v) Anti total-ERKs from Cell-Signaling #9102 and vi) Anti total-ERKs homemade E1B [10]. (PDF 2233 kb) [file 12862_2015_450_MOESM3_ESM.pdf]
