# Supplementary material for: ERK1 and ERK2 present functional redundancy in tetrapods despite higher evolution rate of ERK1
Source: BMC Evol Biol. 2015 Sep 3;15:179. doi: 10.1186/s12862-015-0450-x (PMC4559367; doi:10.1186/s12862-015-0450-x)

Additional file 5

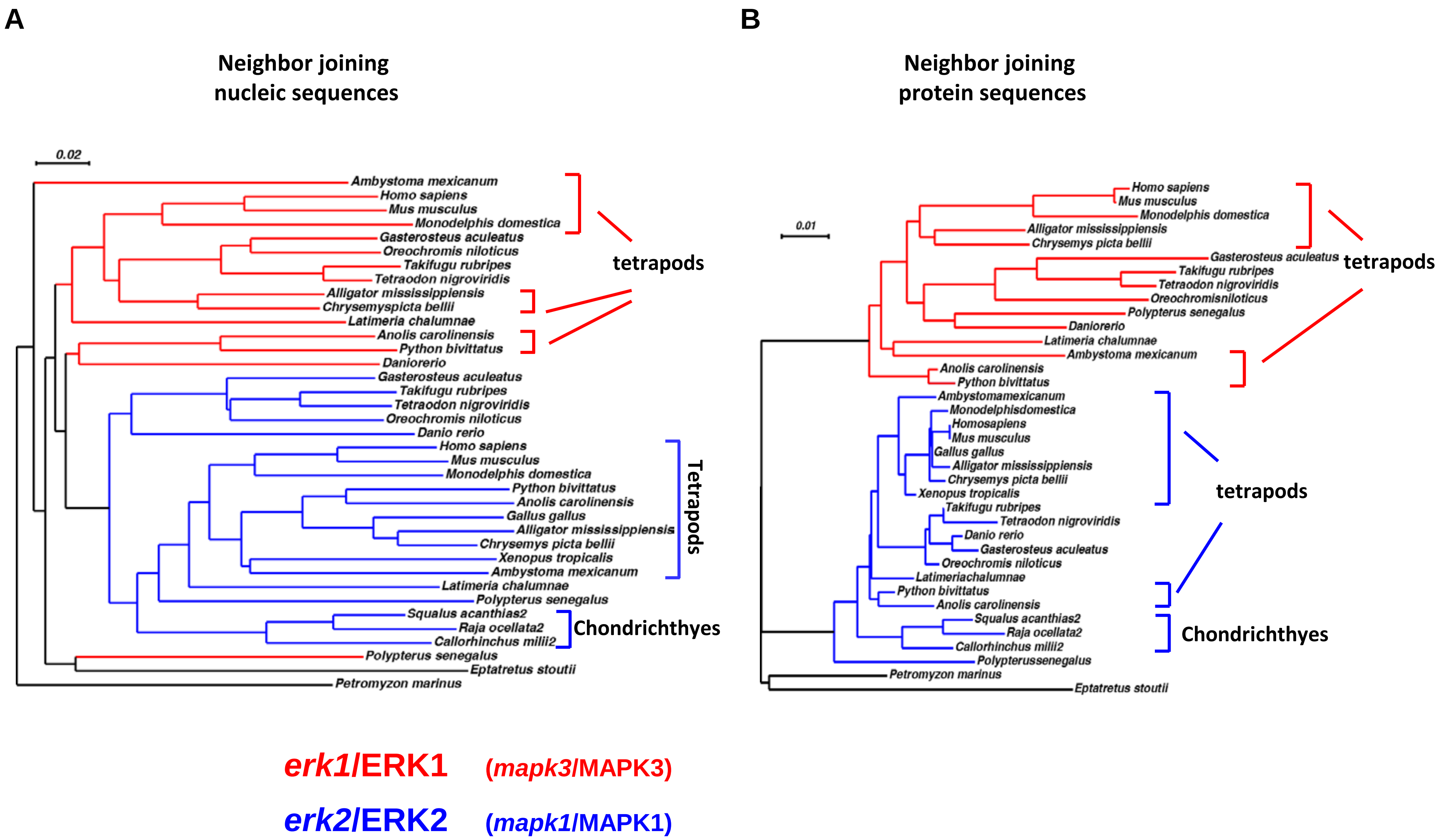

Additional file 5

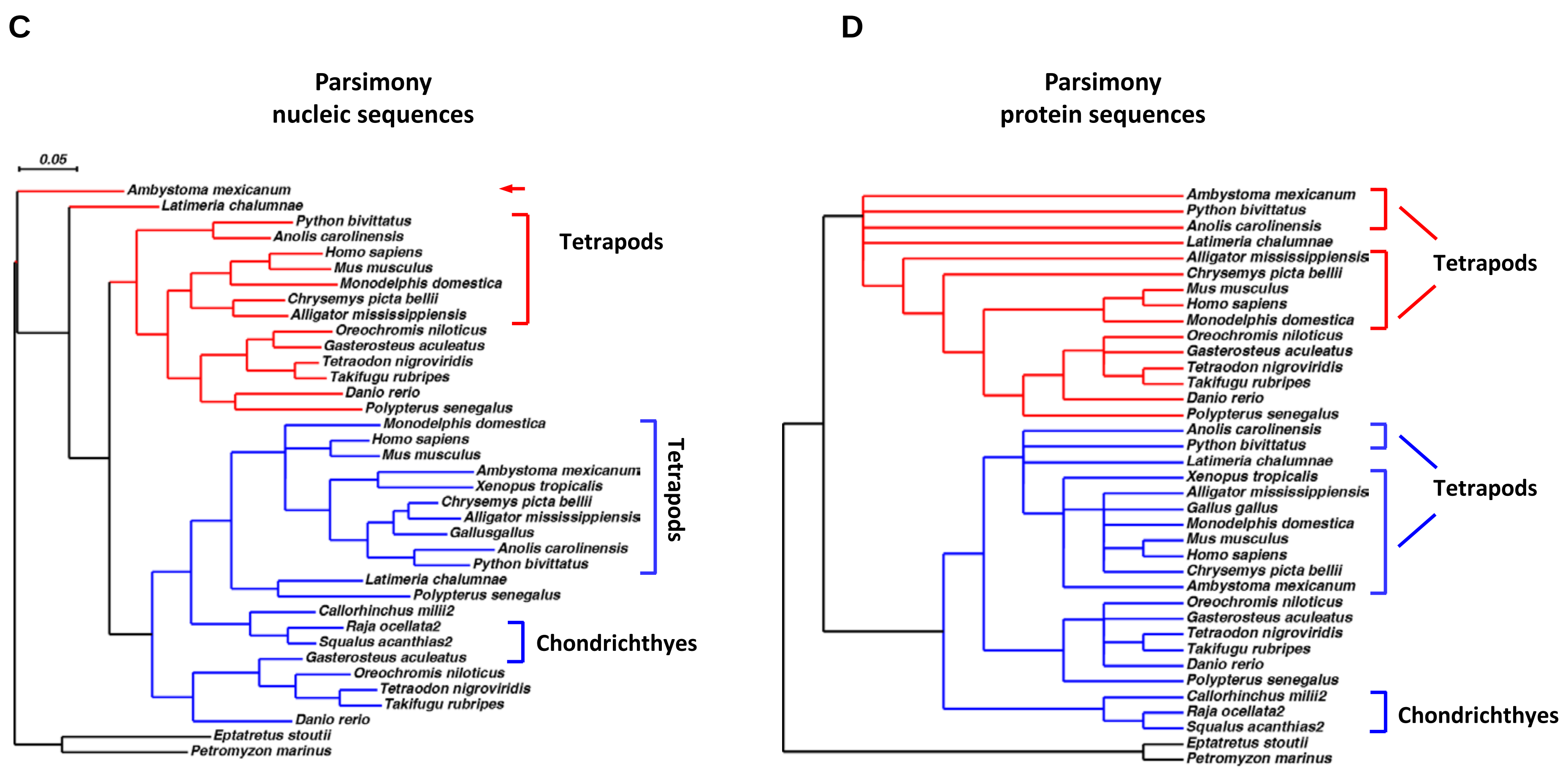

erk1/ERK1 (*mapk3*/MAPK3)

erk2/ERK2 (*mapk1*/MAPK1)

Additional file 5

E

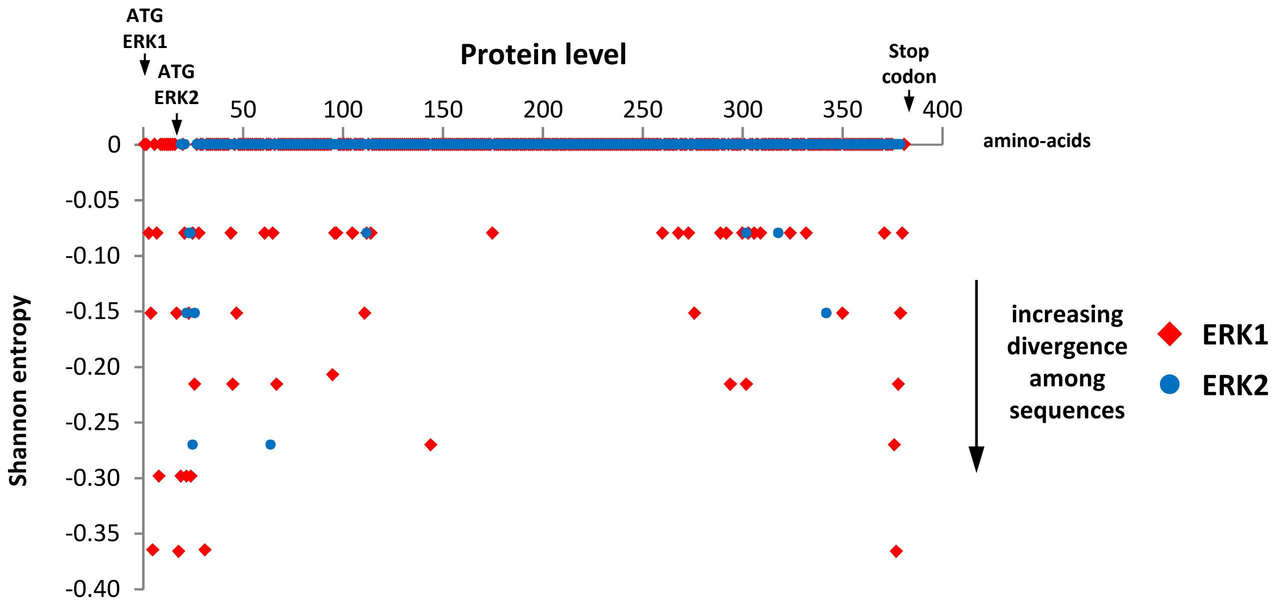

F

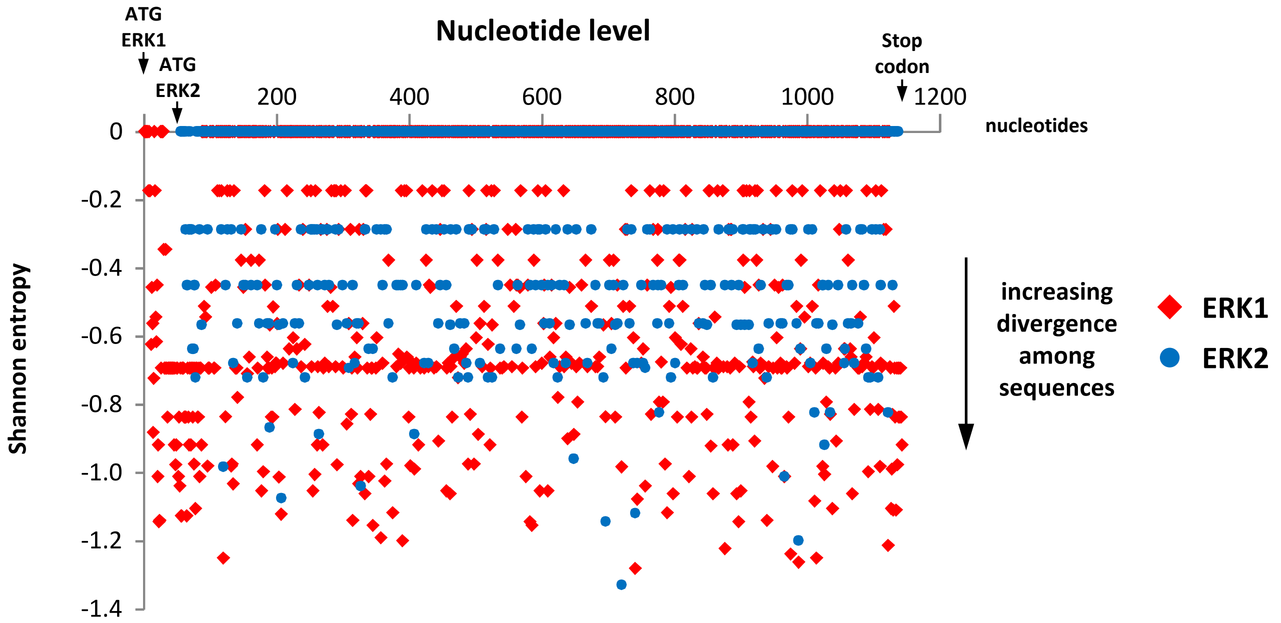

Supplement: Additional file 5: — Phylograms of ERK nucleic or protein sequences in vertebrates and sequence divergences. Phylogenetic analysis of erk nucleotides sequences (A,C) and derived ERK amino acid sequences (B,D) from organisms at key evolutionary nodes in the vertebrate linage by neighbor joining analysis (A,B) and maximum parsimony (C,D). erk1/ERK1 (mapk3/MAPK3, red branches) erk2/ERK2 sequences (mapk1/MAPK1, blue branches). Tetrapods or Chondrichthyes are indicated by arrows or brackets. Accession numbers and common names are listed in Figure 7. (E,F) Shannon entropy for erk sequences. A multiple sequence alignment was conducted on all available full-length ERK1/erk1 and ERK2/erk2 pairs of mammalian sequences (12 pairs) and a .fasta file was constructed for each gene using the conserved domains determined by the alignment. (E) Abscissa: distance from start ATG codon (F) Abscissa: distance from starting mRNA nucleotide. Ordinate: calculated Shannon entropy values for each position, amino-acid (E) and nucleotide (F). Arrows on the side indicate that the lower entropy indicate greater divergences among sequences at that residue position. In the 12 mammals analyzed, ERK1 proteins diverge at 42 positions whereas ERK2 proteins diverge at only 11 positions. Furthermore, without taking into account the extreme N-terminal poly-alanine stretch, the difference is wider: 35 positions diverge among ERK1s versus 4 positions among ERK2s. (PDF 2242 kb) [file 12862_2015_450_MOESM5_ESM.pdf]
