# Supplementary material for: ERK1 and ERK2 present functional redundancy in tetrapods despite higher evolution rate of ERK1
Source: BMC Evol Biol. 2015 Sep 3;15:179. doi: 10.1186/s12862-015-0450-x (PMC4559367; doi:10.1186/s12862-015-0450-x)
Supplement: Additional file 6: — ERK1 and ERK2 protein expression in reptiles. (A to C) presentations as in Additional file 3. (A) Cerebellum samples from lizard (A. Carolinensis), crocodile (C. niloticus) and turtle (T. scripta elegans), control extracts from cattle cortex (Bos Taurus) are loaded on first lane. (B) samples from gecko (T. mauritanica); lizard (A. Carolinensis); snake (T. elegans; mouse NIH3T3 were stimulated for 10 min prior to lysis (lane1) or were stimulated one hour in presence of MEK inhibitor PD184352 (lane2) to block ERK phosphorylation by MEK Kinase. (C) Triton lysis of different lizard extracts (A. Carolinensis) was performed as described in materials and methods, 10 % acrylamide gels were loaded with 15 μg protein. Extract from mouse NIH3T3 fibroblasts (stimulated for one hour with sodium-orthovanadate and 10 % serum) was loaded in lane 1 as control. Upper panel: coomassie staining illustrates overall uniform protein loading of samples despite great differences in expression of major abundant proteins. Median panel: anti total-ERK antibody reveals ERK1 and ERK2 proteins in mouse and only ERK1 protein in all anolis tissues. Lower panel: anti-phospho ERK antibody confirms that only one ERK is expressed in all anolis tissues tested. Note that despite triton lysis and centrifugation of insoluble material, major proteins produce some non-specific binding. (D) Full species names of reptiles whose brains were dissected in the laboratory for this study. (PDF 2230 kb) [file 12862_2015_450_MOESM6_ESM.pdf]

**Additional file 6**

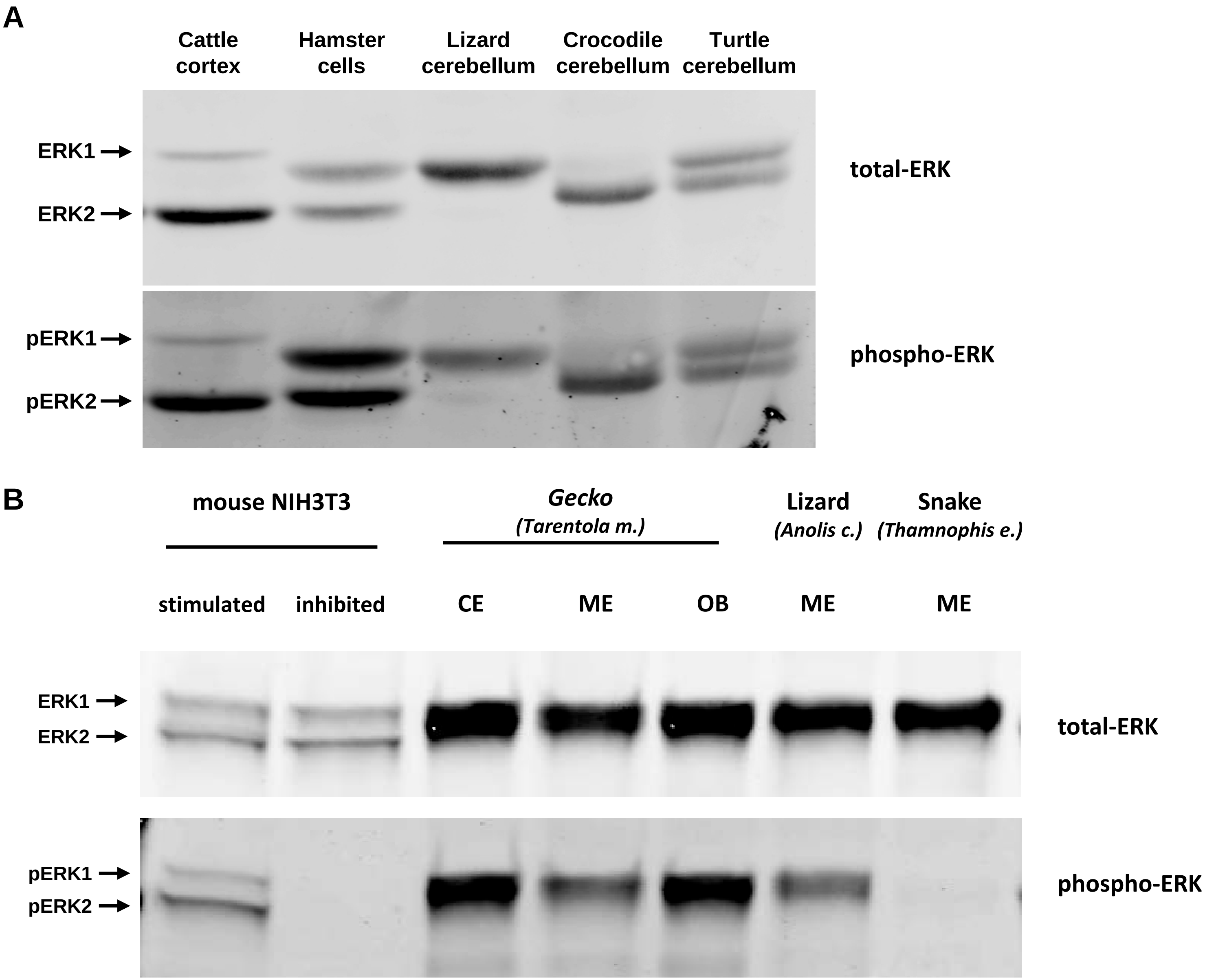

Additional file 6

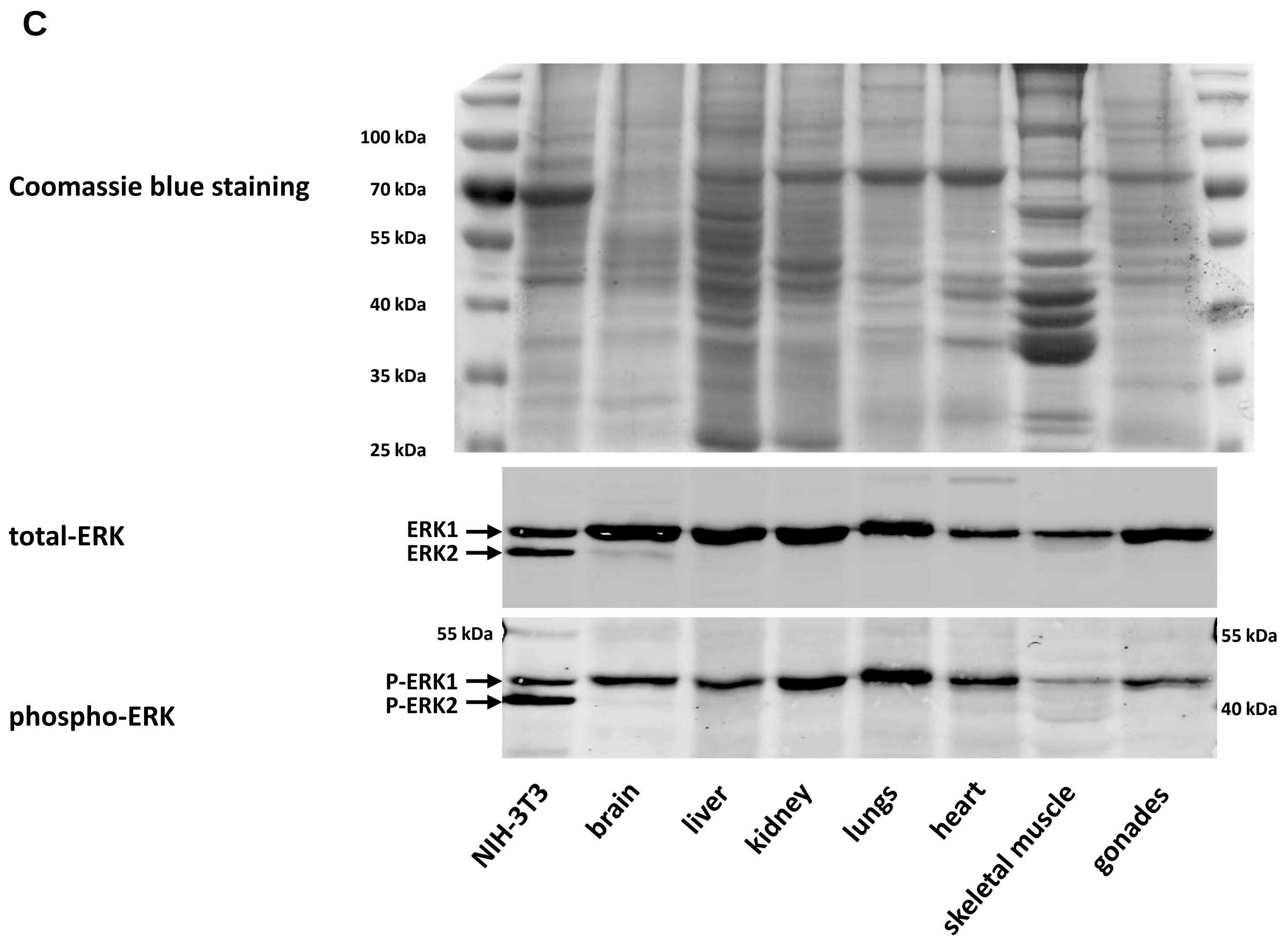

**Additional file 6**

**D**

## Names of reptiles studied

| | |
|---|---|
| **Snakes** | *- Pantherophis guttatus guttatus*<br>*- Pituophis catenifer sayi*<br>*- Lampropeltis triangulum campbelli*<br>*- Thamnophis elegans* |
| **Lizards** | *- Anolis carolinensis*<br>*- Anolis sagrei*<br>*- Podarcis muralis*<br>*- Agama agama* |
| **Gecko** | *- Tarentola mauritanica* |
| **Crocodile** | *- Crocodylus niloticus* |
| **Turtles** | *- Trachemys scripta elegans*<br>*- Emys orbicularis* |
