# Supplementary material for: ERK1 and ERK2 present functional redundancy in tetrapods despite higher evolution rate of ERK1
Source: BMC Evol Biol. 2015 Sep 3;15:179. doi: 10.1186/s12862-015-0450-x (PMC4559367; doi:10.1186/s12862-015-0450-x)
Supplement: Additional file 7: — Animal names and accession numbers of erk cDNA sequences analyzed in the present study. (A) List of vertebrate sequences used in Figure 2A, Figure 2B and Additional Figure 5A-D. ﻿﻿﻿﻿(B) list of 37 mammalian pairs of ERKs whose sequences were used for Fig. 5. (C) list of 12 mammalian pairs of full-length ERKs sequences, used for Additional Figure 5E-F. (D) Correspondence between taxonomic and common names of the animals studied. list of 37 mammalian pairs of ERKs whose sequences were used for Figure S4C and D. list of 12 mammalian pairs of full-length ERKs sequences, used for Figure S7. taxonomic names of animals studied in this work. (PDF 2233 kb) [file 12862_2015_450_MOESM7_ESM.pdf]

Additional figure 7

(A) list of vertebrates whose erk genes are studied

Alligator mississippiensis	ERK1 (MAPK3)	XM_006265720.1	aligator	Tetrapod			
Alligator mississippiensis	ERK2 (MAPK1)	XM_006269319.1	aligator	Tetrapod			
Ambystoma mexicanum	ERK1 (MAPK3)	C0314240	axololt	Tetrapod		database	http://www.ambystoma.org/
Ambystoma mexicanum	ERK2 (MAPK1)	C0317283	axololt	Tetrapod		database	http://www.ambystoma.org/
Anolis carolinensis	ERK1 (MAPK3)	XM_003225001.1	lizard	Tetrapod			
Anolis carolinensis	ERK2 (MAPK1)	XM_003225115.1	lizard	Tetrapod			
Chrysemys picta bellii	ERK1 (MAPK3)	XM_005290182.1	turtle	Tetrapod			
Chrysemys picta bellii	ERK2 (MAPK1)	XM_005281100.1	turtle	Tetrapod			
Gallus gallus	ERK2 (MAPK1)	NM_204150.1	chicken	tetrapod			
Homo sapiens	ERK1 (MAPK3)	NM_002746.2	human	Tetrapod			
Homo sapiens	ERK2 (MAPK1)	NM_138957.3	human	Tetrapod			
Monodelphis domestica	ERK1 (MAPK3)	XM_001364326.1	opossum	Tetrapod			
Monodelphis domestica	ERK2 (MAPK1)	XM_001378172.2	opossum	Tetrapod			
Mus musculus	ERK1 (MAPK3)	NM_011952.2	mouse	Tetrapod			
Mus musculus	ERK2 (MAPK1)	NM_011949.3	mouse	Tetrapod			
Python bivittatus	ERK1 (MAPK3)	XM_007424312.1	snake	Tetrapod			
Python bivittatus	ERK2 (MAPK1)	XM_007422438.1	snake	Tetrapod			
Xenopus tropicalis	ERK2 (MAPK1)	NM_001017127.2	clawed frog	Tetrapod			
Latimeria chalumnae	ERK1 (MAPK3)	XM_006005913.1	coelacanth	Lobe-finned fish			
Latimeria chalumnae	ERK2 (MAPK1)	XM_005990177.1	coelacanth	Lobe-finned fish			
Danio rerio	ERK1 (MAPK3)	NM_201507.1	zebrafish	Ray-finned fishes	Teleost		
Danio rerio	ERK2 (MAPK1)	NM_182888.2	zebrafish	Ray-finned fishes	Teleost		
Oreochromis niloticus	ERK1 (MAPK3)	XM_003453986.2	tilapia	Ray-finned fishes	Teleost		
Oreochromis niloticus	ERK2 (MAPK1)	XM_003444474.2	tilapia	Ray-finned fishes	Teleost		
Takifugu rubripes	ERK1 (MAPK3)	XM_003964469.1	pufferfish	Ray-finned fishes	Teleost		
Takifugu rubripes	ERK2 (MAPK1)	XM_003975069.1	pufferfish	Ray-finned fishes	Teleost		
Polypterus senegalus	ERK1 (MAPK3)	KT324591	bichir	Ray-finned fishes			
Polypterus senegalus	ERK2 (MAPK1)	KT324592	bichir	Ray-finned fishes			
Callorhinchus milii	ERK2 (MAPK1)	XM_007908609	elephant shark	Cartilaginous fish			
Raja ocellata	ERK2 (MAPK1)	KT324593	skate	Cartilaginous fish			
Squalus acanthias	ERK2 (MAPK1)	KT324594	dogfish shark	Cartilaginous fish			
Petromyzon marinus	ERK	ENSPMAG00000009252	lamprey	Jawless vertebrate			
Eptatretus stoutii	ERK	KT324595	hagfish	Jawless vertebrate			

Additional figure 7

(B) list of 37 of mammals whose pairs of erk genes are sequenced at least after first exon

Ailuropoda melanoleuca	ERK1	(MAPK3)	XM_002927335.1	Giant panda
Ailuropoda melanoleuca	ERK2	(MAPK1)	XM_002926299.1	
Bos taurus	ERK1	(MAPK3)	XM_005224919.1	Cattle
Bos taurus	ERK2	(MAPK1)	NM_175793.2	
Bubalus bubalis	ERK1	(MAPK3)	XM_006070498.1	Water buffalo
Bubalus bubalis	ERK2	(MAPK1)	XM_006065600.1	
Callithrix jacchus	ERK1	(MAPK3)	XM_002807415	Common marmoset
Callithrix jacchus	ERK2	(MAPK1)	XM_002743577.2	
Canis lupus familiaris	ERK1	(MAPK3)	NM_001252035.1	Dog
Canis lupus familiaris	ERK2	(MAPK1)	NM_001110800.1	
Capra hircus	ERK1	(MAPK3)	XM_005698000.1	Goat
Capra hircus	ERK2	(MAPK1)	XM_005691765.1	
Chrysochloris asiatica	ERK1	(MAPK3)	XM_006877029.1	Cape golden mole
Chrysochloris asiatica	ERK2	(MAPK1)	XM_006877634.1	
Cricetulus griseus	ERK1	(MAPK3)	XM_003510126.1	Chinese hamster
Cricetulus griseus	ERK2	(MAPK1)	XM_003502846.1	
Equus caballus	ERK1	(MAPK3)	XM_001915525.3	Horse
Equus caballus	ERK2	(MAPK1)	XM_005612382.1	
Felis catus	ERK1	(MAPK3)	XM_006942188.1	Cat
Felis catus	ERK2	(MAPK1)	XM_003994973.2	
Heterocephalus glaber	ERK1	(MAPK3)	XM_004856274.1	Naked mole rat
Heterocephalus glaber	ERK2	(MAPK1)	XM_004843322.1	
Homo sapiens	ERK1	(MAPK3)	NM_002746.2	Human
Homo sapiens	ERK2	(MAPK1)	NM_002745.4	
Jaculus jaculus	ERK1	(MAPK3)	XM_004670246.1	Lesser Egyptian jerboa
Jaculus jaculus	ERK2	(MAPK1)	XM_004670774.1	
Loxodonta africana	ERK1	(MAPK3)	XM_003418924.1	African bush elephant
Loxodonta africana	ERK2	(MAPK1)	XM_003419308.1	
Macaca fascicularis	ERK1	(MAPK3)	XM_005591814.1	Crab-eating macaque
Macaca fascicularis	ERK2	(MAPK1)	XM_005567863.1	
Macaca mulatta	ERK1	(MAPK3)	XM_002802443.1	Rhesus macaque
Macaca mulatta	ERK2	(MAPK1)	XM_001089600.2	
Mesocricetus auratus	ERK1	(MAPK3)	XM_005064413.1	Golden hamster
Mesocricetus auratus	ERK2	(MAPK1)	XM_005077569.1	
Microtus ochrogaster	ERK1	(MAPK3)	XM_005351933.1	Prairie vole
Microtus ochrogaster	ERK2	(MAPK1)	XM_005370009.1	
Monodelphis domestica	ERK1	(MAPK3)	XM_001364326.2	Gray short-tailed opossum
Monodelphis domestica	ERK2	(MAPK1)	XM_001378172.3	
Mus musculus	ERK1	(MAPK3)	NM_011952.2	House mouse
Mus musculus	ERK2	(MAPK1)	XM_006522147.1	
Mustela putorius furo	ERK1	(MAPK3)	XM_004774037.1	Ferret
Mustela putorius furo	ERK2	(MAPK1)	XM_004742524.1	
Myotis brandtii	ERK1	(MAPK3)	XM_005881979.1	Brandt's bat
Myotis brandtii	ERK2	(MAPK1)	XM_005875499.1	
Myotis lucifugus	ERK1	(MAPK3)	XM_006103356.1	Little brown bat
Myotis lucifugus	ERK2	(MAPK1)	XM_006099510.1	
Octodon degus	ERK1	(MAPK3)	XM_004622941.1	Degu
Octodon degus	ERK2	(MAPK1)	XM_004627959.1	
Odobenus rosmarus divergens	ERK1	(MAPK3)	XM_004397172.1	Walrus
Odobenus rosmarus divergens	ERK2	(MAPK1)	XM_004400515.1	
Otolemur garnettii	ERK1	(MAPK3)	XM_003795779.1	Northern greater galago
Otolemur garnettii	ERK2	(MAPK1)	XM_003803280.1	
Ovis aries	ERK1	(MAPK3)	XM_004021216.1	Sheep
Ovis aries	ERK2	(MAPK1)	XM_004017677.1	
Panthera tigris altaica	ERK1	(MAPK3)	XM_007093492.1	Siberian tiger
Panthera tigris altaica	ERK2	(MAPK1)	XM_007091093.1	
Papio anubis	ERK1	(MAPK3)	XM_003916743.1	Olive baboon
Papio anubis	ERK2	(MAPK1)	XM_003905274.1	
Peromyscus maniculatus bairdii	ERK1	(MAPK3)	XM_006977116.1	deer mouse
Peromyscus maniculatus bairdii	ERK2	(MAPK1)	XM_006993033.1	
Physeter catodon	ERK1	(MAPK3)	XM_007107444.1	Sperm whale
Physeter catodon	ERK2	(MAPK1)	XM_007130607.1	
Pteropus alecto	ERK1	(MAPK3)	XM_006914227.1	Black flying fox
Pteropus alecto	ERK2	(MAPK1)	XM_006908723.1	
Rattus norvegicus	ERK1	(MAPK3)	NM_017347.2	Brown rat
Rattus norvegicus	ERK2	(MAPK1)	NM_053842.1	
Sarcophilus harrisii	ERK1	(MAPK3)	XM_003762077.1	Tasmanian devil
Sarcophilus harrisii	ERK2	(MAPK1)	XM_003762739.1	
Sorex araneus	ERK1	(MAPK3)	XM_004615845.1	Common shrew
Sorex araneus	ERK2	(MAPK1)	XM_004607449.1	
Spermophilus tridecemlineatus	ERK1	(MAPK3)	XM_005323935.1	Thirteen-lined ground squirrel
Spermophilus tridecemlineatus	ERK2	(MAPK1)	XM_005334566.1	
tupaia chinensis	ERK1	(MAPK3)	XM_006148278.1	Tree shrew
Tupaia chinensis	ERK2	(MAPK1)	XM_006140221.1	

Additional figure 7

(C) list of 12 of mammals whose pairs of erk genes are fully sequenced

Canis lupus familiaris	ERK1	(MAPK3)	NM_001252035.1	Dog
Canis lupus familiaris	ERK2	(MAPK1)	NM_001110800.1	
Homo sapiens	ERK1	(MAPK3)	NM_002746.2	Human
Homo sapiens	ERK2	(MAPK1)	NM_002745.4	
Mus musculus	ERK1	(MAPK3)	NM_011952.2	Mouse
Mus musculus	ERK2	(MAPK1)	XM_006522147.1	
Rattus norvegicus	ERK1	(MAPK3)	NM_017347.2	Rat
Rattus norvegicus	ERK2	(MAPK1)	NM_053842.1	
Bos taurus	ERK1	(MAPK3)	XM_005224919.1	Cattle
Bos taurus	ERK2	(MAPK1)	NM_175793.2	
Monodelphis domestica	ERK1	(MAPK3)	XM_001364326.2	Opossum
Monodelphis domestica	ERK2	(MAPK1)	XM_001378172.3	
Otolemur garnettii	ERK1	(MAPK3)	XM_003795779.1	Northern greater galago
Otolemur garnettii	ERK2	(MAPK1)	XM_003803280.1	
Papio anubis	ERK1	(MAPK3)	XM_003916743.1	Baboon
Papio anubis	ERK2	(MAPK1)	XM_003905274.1	
Odobenus rosmarus divergens	ERK1	(MAPK3)	XM_004397172.1	Walrus
Odobenus rosmarus divergens	ERK2	(MAPK1)	XM_004400515.1	
Octodon degus	ERK1	(MAPK3)	XM_004622941.1	Degu
Octodon degus	ERK2	(MAPK1)	XM_004627959.1	
Heterocephalus glaber	ERK1	(MAPK3)	XM_004856274.1	Naked mole rat
Heterocephalus glaber	ERK2	(MAPK1)	XM_004843322.1	
Chrysochloris asiatica	ERK1	(MAPK3)	XM_006877029.1	Cape golden mole
Chrysochloris asiatica	ERK2	(MAPK1)	XM_006877634.1	

Additional figure 7

(D) Nomenclature used for tested animal samples

	Common names of animals	Taxonomic names of animals
Fig. 1	Mouse	*Mus musculus*
	Hagfish	*Eptatretus stoutii*
	Cattle	*Bos taurus*
	Spiny dogfish	*Squalus acanthias*
	Smaller spotted catshark	*Scyliorhinus canicula*
	Lamprey	*Petromyzon marinus*
	Winter skate	*Raja ocellata*
	Bichir	*Polypterus senegalus*
Fig. 3A	Cattle	*Bos taurus*
	Corn snake	*Pantherophis guttatus guttatus*
	Bull snake	*Pituophis catenifer sayi*
	Green anole	*Anolis carolinensis*
	Brown anole	*Anolis sagrei*
	crocodile	*Crocodylus niloticus*
	Red-eared slider	*Trachemys scripta elegans*
	European pond turtle	*Emys orbicularis*
	Mouse	*Mus musculus*
Fig. 3B	Mouse	*Mus musculus*
	Green anole	*Anolis carolinensis*
	Brown anole	*Anolis sagrei*
fig. S3	European sea bass	*Dicentrarchus labrax*
	African jewelfish	*Hemichromis bimaculatus*
	Bichir	*Polypterus senegalus*
	African clawed frog	*Xenopus laevi*
	Axolotl	*Ambiostoma mexicanum*
	Chicken	*Gallus gallus*
	Greater Rhea	*Rhea americana*
	European rabbit	*Oryctolagus cuniculus*
	Cattle	*Bos taurus*
	Opossum	*Monodelphis Domestica*
	Chinese hamster	*Cricetulus griseus*
fig. S4	Cattle	*Bos taurus*
	Chinese hamster	*Cricetulus griseus*
	Green anole	*Anolis carolinensis*
	Crocodile	*Crocodylus niloticus*
	Red-eared slider	*Trachemys scripta elegans*

Mouse	*Mus musculus*
Common gecko	*Tarentola mauritanica*
Green anole	*Anolis carolinensis*
Garter snake	*Thamnophis elegans*
